# Unraveling nitrogen metabolism, cold and stress adaptation in polar *Bosea* sp. PAMC26642 through comparative genome analysis

**DOI:** 10.3389/fmicb.2024.1505699

**Published:** 2025-01-24

**Authors:** Anamika Khanal, So-Ra Han, Jun Hyuck Lee, Tae-Jin Oh

**Affiliations:** ^1^Genome-Based Bio-IT Convergence Institute, Asan, Republic of Korea; ^2^Bio Big Data-Based Chungnam Smart Clean Research Leader Training Program, SunMoon University, Asan, Republic of Korea; ^3^Department of Life Science and Biochemical Engineering, Graduate School, SunMoon University, Asan, Republic of Korea; ^4^Division of Life Sciences, Korea Polar Research Institute, Incheon, Republic of Korea; ^5^Department of Pharmaceutical Engineering and Biotechnology, SunMoon University, Asan, Republic of Korea

**Keywords:** *Bosea* sp., cold adaptation, comparative genomics, nitrogen metabolism, Gram-negative bacteria

## Abstract

Nitrogen metabolism, related genes, and other stress-resistance genes are poorly understood in *Bosea* strain. To date, most of the research work in *Bosea* strains has been focused on thiosulfate oxidation and arsenic reduction. This work aimed to better understand and identify genomic features that enable thiosulfate-oxidizing lichen-associated *Bosea* sp. PAMC26642 from the Arctic region of Svalbard, Norway, to withstand harsh environments. Comparative genomic analysis was performed using various bioinformatics tools to compare *Bosea* sp. PAMC26642 with other strains of the same genus, emphasizing nitrogen metabolism and stress adaptability. During genomic analysis of *Bosea* sp. PAMC26642, assimilatory nitrogen metabolic pathway and its associated enzymes such as nitrate reductase, NAD(P)H-nitrite reductase, ferredoxin-nitrite reductase, glutamine synthetase, glutamine synthase, and glutamate dehydrogenase were identified. In addition, carbonic anhydrase, cyanate lyase, and nitronate monooxygenase were also identified. Furthermore, the strain demonstrated nitrate reduction at two different temperatures (15°C and 25°C). Enzymes associated with various stress adaptation pathways, including oxidative stress (superoxide dismutase, catalase, and thiol peroxidase), osmotic stress (OmpR), temperature stress (Csp and Hsp), and heavy metal resistance, were also identified. The average Nucleotide Identity (ANI) value is found to be below the threshold of 94-95%, indicating this bacterium might be a potential new species. This study is very helpful in determining the diversity of thiosulfate-oxidizing nitrate-reducing bacteria, as well as their ability to adapt to extreme environments. These bacteria can be used in the future for environmental, biotechnological, and agricultural purposes, particularly in processes involving sulfur and nitrogen transformation.

## 1 Introduction

Nitrogen metabolism and its regulation have been studied across various ecosystems, including the polar regions (Arctic/Antarctic) by various microorganisms such as Cyanobacteria (*Nitrosospira*, *Nitrosomonas*, *Nostoc*, and *Anabaena*) (Hayashi et al., 2020; Magalhães et al., 2014; Makhalanyane et al., 2015; Fernández-Valiente et al., 2001), Actinobacteria (*Streptomyces and Frankiniaceae*) (De Scally et al., 2016; Papale et al., 2018), and Proteobacteria (*Burkholderiales*) (Garrido-Benavent et al., 2020). Nitrogen metabolism in prokaryotes involves a complex interplay between transporter proteins, signaling proteins, and transcriptional regulators. Furthermore, they also involve the coordinated expression of enzymes that utilize extracellular nitrogen sources and intracellular biosynthesis of nitrogen-containing compounds (Harper et al., 2008; Merrick and Edwards, 1995). Despite these insights, there remains a significant gap in understanding how bacteria meet their nitrogen requirements. This gap is particularly evident in extreme environments, such as the polar regions. One such bacterium is *Bosea* sp. (specifically, *Bosea* sp. PAMC26642) reported by Kim et al. (2013) whose ability to survive and thrive in cold and nutrient-poor conditions could provide valuable information on microbial adaptation.

The genus *Bosea* was first reported by Das et al. in 1996. It belongs to the order *Hyphomicrobiales* and the family *Boseaceae* (Das and Mishra, 1996; Hördt et al., 2020). In addition to that, *Bosea* species have been isolated from various environments including Arctic lichen (*Bosea* sp. PAMC26642) (Kim et al., 2013), agricultural soils (Das et al., 1996), hospital water systems (La Scola et al., 2003), lakes (Albert et al., 2019), anaerobic digester sludge (Ouattara et al., 2003), root nodules of legumes (De Meyer and Willems, 2012), and pyrite rock (Walczak et al., 2018). *Bosea* strains were reported to have arsenite-, sulfide-, and antimonite-oxidizing abilities (Walczak et al., 2018; Lu et al., 2018). *Bosea* strains are mostly studied for the remediation purpose of metalloids such as arsenic and antimony removal/transformation (Lu et al., 2018; Xiang et al., 2022). Although *Bosea* species have been studied across different environments and for various purposes, studies focusing on their nitrogen metabolism remain limited. In particular, the nitrogen metabolism of *Bosea* sp. PAMC26642 has not yet been explored.

The main aim of this study was to have a deeper understanding of the bacteria *Bosea* sp. PAMC26642; to conduct the genome analysis of *Bosea* sp. PAMC26642 strain; furthermore, to compare the strain with other *Bosea* species from the same genus with the use of various bioinformatics tools and software; to determine the unique genes/enzymes that allow them to adapt to the extreme environment; and to focus primarily on nitrogen metabolic enzymes and the associated nitrate assimilation pathway. The findings from the study will lay the foundation for leveraging cold-adapted microorganisms like *Bosea* sp. PAMC26642 to address environmental challenges and promote ecosystem resilience in a changing climate. Furthermore, the potential bacteria can be used in the future for environmental, agricultural, and biotechnological purposes.

## 2 Materials and methods

### 2.1 Isolation source, sequencing, and genome information

*Bosea* sp. PAMC26642 was isolated from the Arctic Lichen *Stereocaulon* sp., collected in Svalbard, Norway (78°55′N, 11°56′E), by the Korea Polar Research Institute (KOPRI, Incheon, Republic of Korea). The detailed procedure of the isolation of *Bosea* sp. PAMC26642 was reported by Kim et al. (2013). After the collection of lichen, a segment of the lichen thallus was excised with sterile scissors or knife and subjected to vertexing for 10 min in sterilized 0.85% NaCl solution, which was subsequently discarded, and the procedure was repeated two times. Afterward, the tissue was further disrupted using a mortar in the same NaCl solution. After the washing steps, the disrupted tissue was spread onto Bennett’s vitamin agar, comprising 10.0 g D-glucose, 1.0 g yeast extract, 2.0 g peptone, 1.0 g beef extract, 1.0 mL vitamins, 1.0 L distilled water, and 16.0 g agar. The plates were incubated at 28°C for 15 to 21 days, allowing for the growth of bacterial colonies. Subsequently, the colonies were subcultured three times to isolate a pure culture. The final pure culture of *Bosea* sp. PAMC26642 was preserved at −80°C in 20% glycerol. After storage, the culture was utilized by selecting the appropriate medium and temperature according to the experimental design.

For complete genome sequencing, genomic DNA was extracted from *Bosea* sp. PAMC26642, using a QIAamp DNA Mini Kit (Qiagen Inc., Valencia, CA), determined the quantity and purity using an Agilent 2,100 Bioanalyzer (Agilent Technologies, Santa Clara, CA). Genome sequencing was performed using PacBio RS II single-molecule real-time (SMRT) sequencing technology (Pacific Biosciences, Menlo Park, CA). SMRTbell library inserts (10 kb) were sequenced using SMRT cells. Raw sequence data were generated from 98,259 reads and 1,308,437,307 bp that were assembled *de novo* by using the hierarchical genome-assembly process (HGAP) protocol (Chin et al., 2013) and RS HGAP Assembly 2 in SMRT analysis version 2.3 software (Pacific Biosciences; https://github.com/PacificBiosciences/SMRT-Analysis). The complete genome sequence has been deposited at GenBank/EMBL/DDBJ under the accession numbers CP014301 and CP014302, and the complete genome sequence of *Bosea* sp. PAMC26642 has already been reported by Kang et al. (2016).

### 2.2 Functional annotation and comparative genome analysis

A total of 11 strains of *Bosea* with a complete genome sequence, including strain of interest, *Bosea* sp. PAMC26642 were retrieved from the NCBI nucleotide database^1^ on 30 August 2023. Functional annotation of the genome of our strain and reference strains was carried out by Rapid Annotation using a Subsystem Technology (RAST) server. The RAST server enables the identification and annotation of genes along with their associated functions as well as coding DNA sequences (CDSs) (Aziz et al., 2008) available at https://rast.nmpdr.org on 14 February 2024. Furthermore, *Bosea* sp. PAMC26642 as well as other reference strains were mined for the presence of genes/proteins having roles in nitrogen metabolism, stress resistance, and cold adaptation.

### 2.3 Phylogenetic comparison

A total of 11 strains of *Bosea* with a complete genome sequence, including our strain, were retrieved from the NCBI nucleotide database (see text footnote 1) on 30 August 2023. A phylogenetic tree was constructed using 16S rRNA sequences of the complete genomes of *Bosea* strains together with other *Bosea* strains obtained from the NCBI. These 16S rRNA sequences were aligned using MUSCLE, MEGA 11, and a neighbor-joining method (Edgar, 2004a; Edgar, 2004b; Tamura et al., 2021; Qi et al., 2004; Felsenstein, 1992; Tamura et al., 2004). A maximum composite likelihood model was used to construct the phylogenetic tree. Branch numbers represent percentages of bootstrap values in 1000 sampling replicates. Similarly, a phylogenetic tree based on housekeeping genes such as *dnaK*, *recA*, *gyrB*, and *trpB* was also prepared.

### 2.4 Genome-based taxonomic analysis by TYGS and average nucleotide identity

The genomic sequence of *Bosea* sp. PAMC26642 strain was uploaded to a free bioinformatics platform, TYGS^2^ for the genome-based taxonomic analysis, accessed on 4 January 2024 (Meier-Kolthoff and Göker, 2019). The phylogenetic tree was reconstructed using FastME 2.1.6.1, including SPR post-processing from the GBDP (Lefort et al., 2015). Branch support was inferred from 100 pseudo-bootstrap replicates each. ANI is used to compare the genomic similarity between two microbial genomes. It is very important for species identification as it provides a high-resolution metric for species delineation, especially in bacteria and archaea. Furthermore, ANI provides a more robust, objective measure and detects even small genomic differences. It allows for fine-scale distinctions between closely related species or strains. ANI values were derived from the ANI tool^3^ (Yoon et al., 2017). Moreover, the genome-to-genome distance calculation web server^4^ (Lee et al., 2016). OrthoANI values were calculated using the Orthologous Average Nucleotide Identity Tool (OAT) software (Lee et al., 2016).

### 2.5 Multiple sequence alignment

From the assembled genome, sequences of *Bosea* sp. PAMC26642 were taken, and the nitrate assimilatory genes were predicted, and annotated by BLASTP. The protein sequences were searched against the Nr and Swiss-Prot databases to find the sequences with the most identities for multiple sequence alignments. Multiple sequence alignments of all the proteins were conducted using Clustal Omega (Sievers and Higgins, 2018) and were subjected to ESPript 3^5^ (Robert and Gouet, 2014). To gain a better understanding of the gene’s function and evolution, the protein sequences were subjected to domain analysis using the InterPro database https://www.ebi.ac.uk/interpro/ (provides functional analysis of proteins by classifying them into families and predicting domains and important sites) (Blum et al., 2021).

### 2.6 Nitrogen metabolism and putative 3D structure modeling

The pathway for nitrogen metabolism in the strain was studied with the KEGG database. 3D structures of these analyzed proteins were predicted by the online program PHYRE 2.0 Server^6^ in the intensive mode (Kelley et al., 2015) accessed on 14 September 2023. Protein sequences were used to search against the ExPDB template library. Sequences with the most identities were used for model generation. All 3D images were generated and colored by rainbow from N to C terminals (Bienfait and Ertl, 2013).

### 2.7 Bacterial growth and nitrate reduction assay

For the wet-lab experiment standardized, high-quality culture media and reagents were used. All the instruments were calibrated regularly according to the manufacturer’s instructions and for precise measurement. The isolated strains were cultured in R2A broth (MB cell Ltd., Seoul, Republic of Korea) at three different temperatures at 15°C, 25°C, and 37°C to check their growth. In addition to that, the strain was also tested for their growth in nitrate broth containing peptone (5 gm), meat extract (3 gm), and KNO_3_ (1 gm) with pH 7.0. A nitrate reduction assay was performed based on the bacterial ability to reduce nitrate to nitrite by using a standard operating procedure. The presence of nitrite can be detected with specific reagents such as reagent A (sulfanilic acid 8 gm/l, glacial acetic acid 286 mL/L, and demineralized water 714 mL/L) and reagent B (glacial acetic acid 286 mL/L, N, N-dimethyl-1-napthaylamine 6 mL/L, and demineralized water 714 mL/L), which produce a color change. For confirmation of nitrate reduction, zinc dust was also added. For quality control measures, both positive and negative controls were used. The strain *Bosea* sp. PAMC26642 (test organism) was cultured in nitrate broth, including an abiotic control without any microorganisms and positive control with *Escherichia coli* strain at three different temperatures for 2 days at 15°C, 25°C, and 37°C; 1 mL of bacterial culture from different temperatures was measured by using a spectrophotometer (Biochrome, Libra S35PC, Cambridge, UK). A uniform OD_600_ having a consistent bacterial cell density was taken in test tubes. A few drops of reagents A and B were added. The change in color to red from a colorless solution was monitored. For confirmation, a pinch of zinc dust was added to the tube with reagents A and B. All the experiments were performed in triplicates and are reproducible.

### 2.8 Proteins that are involved in different stress adaptation mechanisms

The complete genome sequence of all 11 strains, including our strain, after annotation from RAST analysis, was also compared in terms of genes that are involved in different stress adaptation mechanisms such as oxidative stress, heavy metal resistance, and salt stress.

## 3 Results and discussion

### 3.1 Complete genome information of *Bosea* sp. strain available on NCBI database

**Figure 1:**
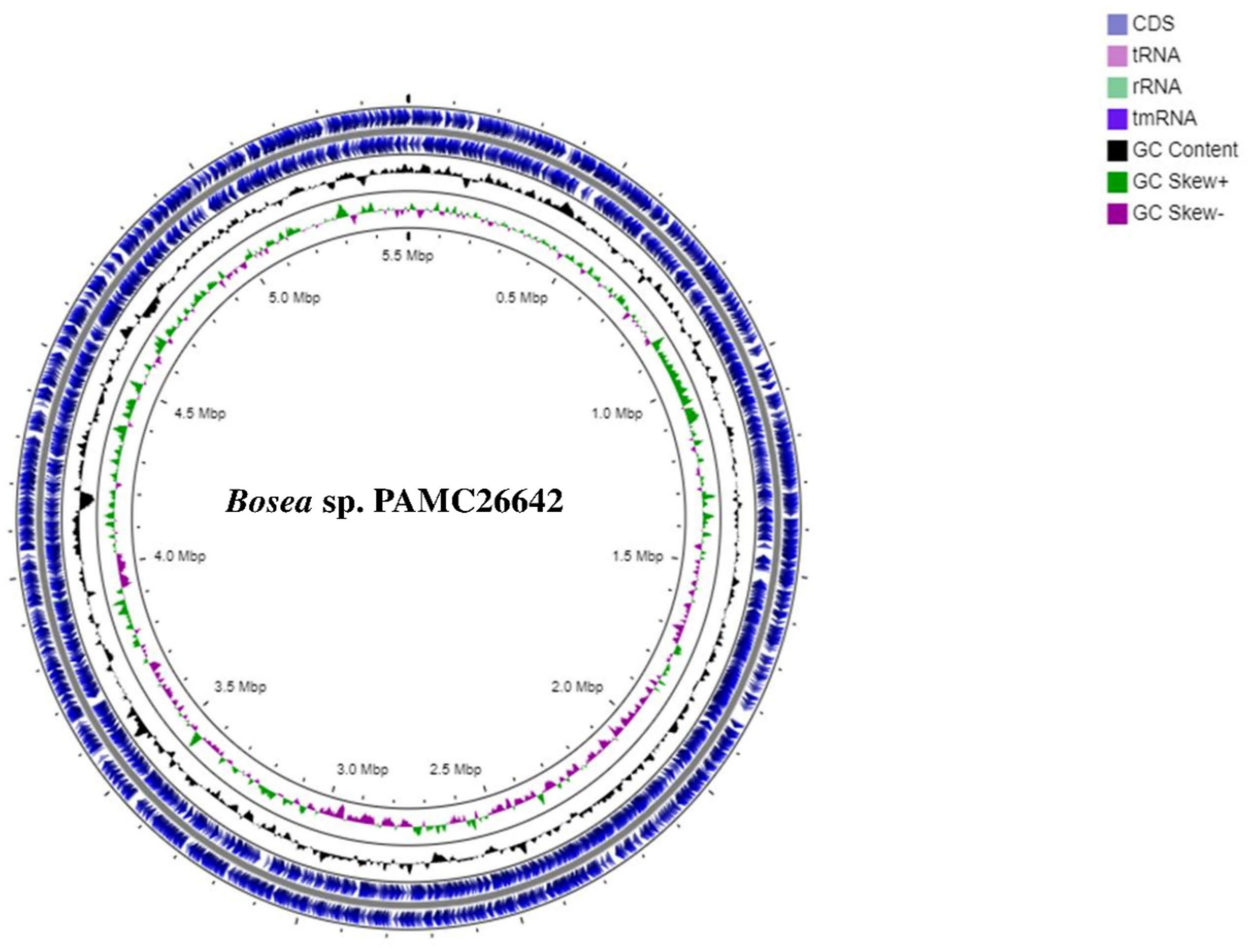
Circular map of *Bosea* sp. PAMC26642. Circular representation of genome with basic features including CDS and tRNA distributions, rRNA, tmRNA, GC content, GC Skew+, and GC Skew-from the outer circle to the inner circle.

**Figure 2:**
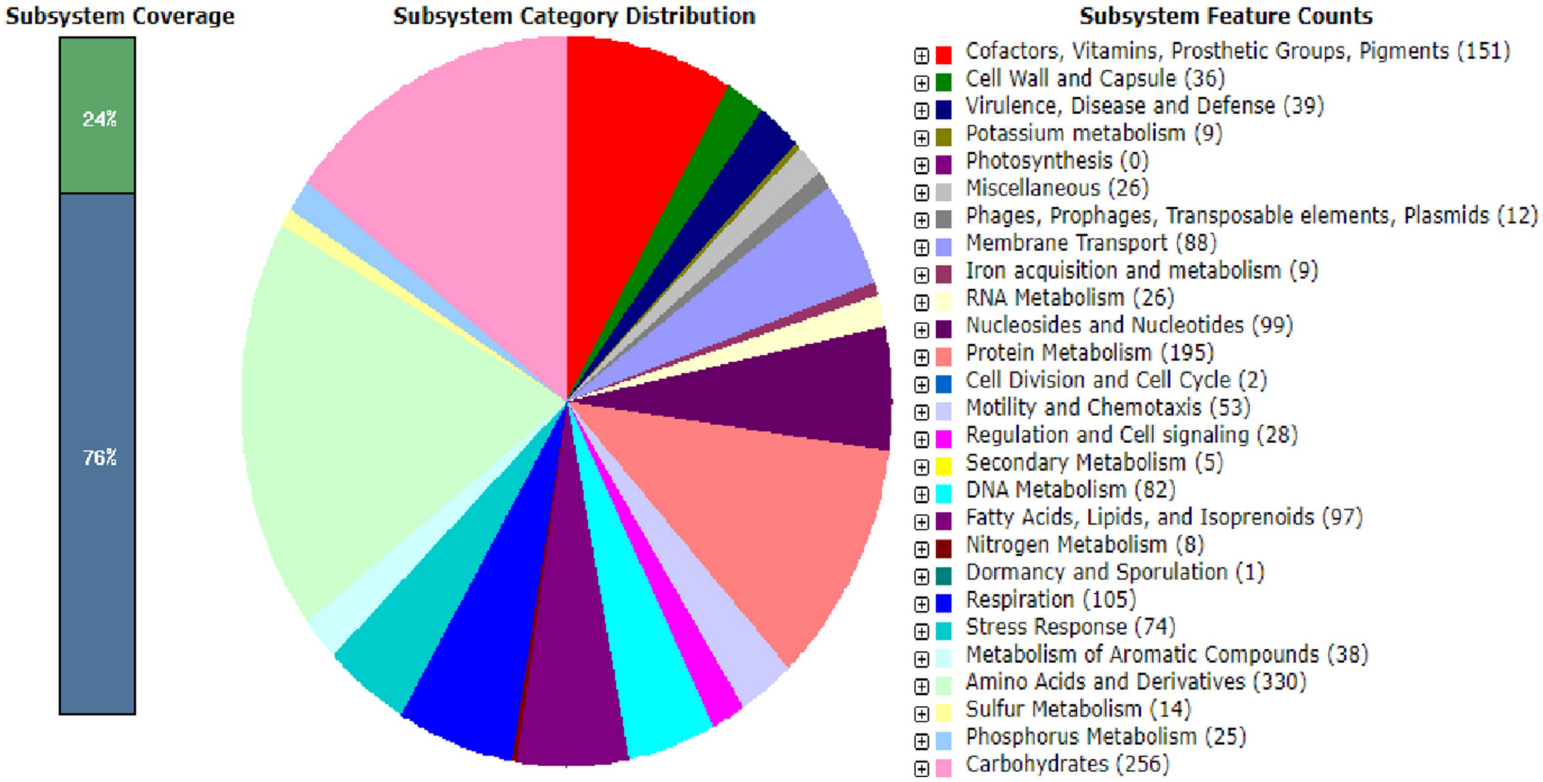
Subsystem distribution based on the RAST SEED analysis of *Bosea* sp. PAMC26642. The number of protein-coding genes (in parenthesis) are predicted to be involved in that cellular process.

A circular map of *Bosea* sp. PAMC26642 and a subsystem distribution based on the RAST SEED analysis of *Bosea* sp. PAMC26642 are shown in Figures 1, 2, respectively. *Bosea* sp. PAMC26642 and other strains of *Bosea* were compared. The general genome features and genomic information for all 11 strains are summarized in Tables 1, 2, respectively. All the compared strains have high GC content and are nearly similar.

**Table 1:** General information and isolation sources of all the *Bosea* strains having a complete genome available in the NCBI database to date.

Organism/name	Strain	Isolate information Isolation source	Isolation country	Geographic location	Host	Sample type	Collection date	Reference
*Bosea* sp.	PAMC26642*	Lichen	Norway	Svalbard	Stereocaulon sp.	Single cell	2014	Kang et al. (2016)
*Bosea* sp.	ANAM02	N/A	Japan	Japan	*Callyspongia*	N/A	2015	-
*Bosea* sp.	AS-1	Soil (Mine)	China	China, Hunan	N/A	N/A	2015	Lu et al. (2018)
*Bosea* sp.	F3-2	Rhizosphere soil	China	China, Guangxi	Soybean	Soil	2017	Zhang et al. (2019)
*Bosea* sp.	RAC05	Algal phycosphere	USA	USA, WA, Seattle	N/A	Microbial isolate	2014	-
*Bosea* sp.	Tri-49	Root nodules	Russia	Russia Baikal Lake region	*Oxytropis triphylla*	Cell culture	2015	-
*Bosea vaviloviae*	Vaf18	N/A	Russia	Russia North, Ossetia, Caucasus	*Vavilovia formosa*	Type strain	2012	-
*Bosea* NBC-00550	NBC_00550	Soil	Denmark	Denmark, Lyngby	N/A	N/A	2016	Alvarez-Arevalo et al. (2023)
*Bose vestrisii*	A18/4–2	Root nodules	Russia	Russia, Altai region	N/A	Cell culture	2020	-
*Bosea* sp. REN20	REN20	Baijiu mash	China	China	N/A	Cell culture	2021	-
*Bosea vaviloviae*	685	Root nodules	Russia	Russia, Kamchatka peninsula	*Astragalus umbellatus*	Cell culture	2016	-

“*“*” refers to lichen-associated bacteria isolated from the Arctic. “-” refers to the sequence available but not published.

**Table 2:** Genomic information of all *Bosea* strains.

Organism strain name	GC %	Genome total length	No of chromosome	No of plasmid	rRNA genes	tRNA genes
*Bosea* sp. PAMC26642*	65	5.51	1	1	3	49
*Bosea* sp. ANAM02	66	5.64	1	1	6	64
*Bosea* sp. AS-1	66	5.83	1	2	6	50
*Bosea* sp. F3-2	65.5	7.04	1	2	9	52
*Bosea* sp. RAC05	67.5	5.62	1	1	6	73
*Bosea* sp. Tri-49	66	6.45	1	1	6	49
*Bosea vavilovia* strain Vaf18	65.5	6.71	1	1	6	49
*Bosea* NBC-00550	65.5	6.26	1	1	6	50
*Bosea vestrisii* A18/4–2	66	6.30	1	1	6	48
*Bosea* sp. REN20	66	5.56	1	-	3	46
*Bosea vaviloviae* 685	65.5	6.71	1	1	6	49

“*“*” refers to lichen-associated bacteria isolated from the Arctic.

### 3.2 Phylogenetic analysis of 16S rRNA and housekeeping genes

**Figure 3:**
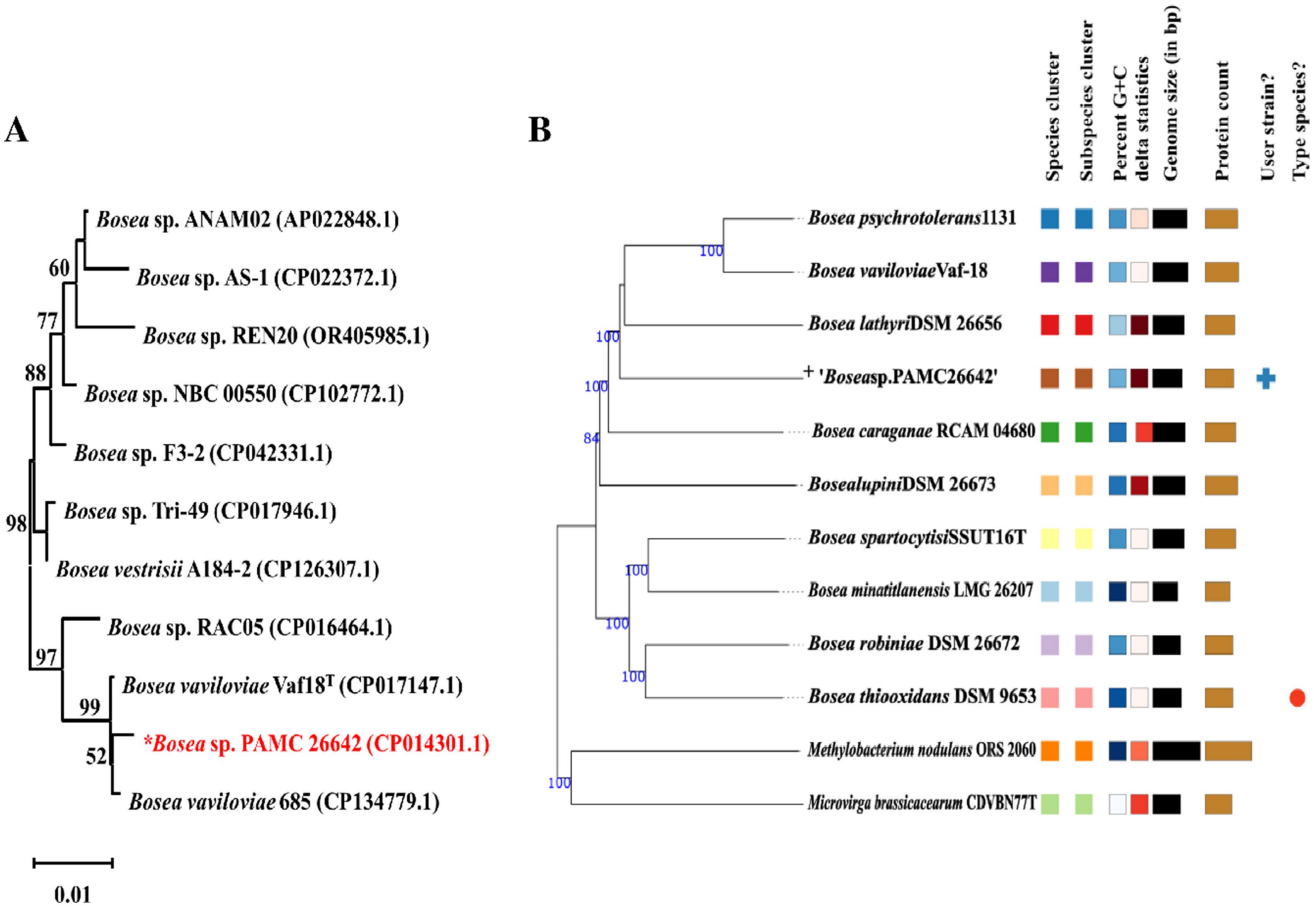
**(A)** Neighbor-joining phylogenetic trees of 16S rRNA gene of *Bosea* sp. without an outgroup. Percentages in the bootstrap test are from 1,000 replicates. **(B)** Phylogenomic tree based on genome sequences in the TYGS tree inferred with FastME 2.1.6.1 from GBDB. The branch lengths are scaled in terms of the GBDP distance formula d5. “+” denote *Bosea* sp. PAMC26642. Different colors are provided to indicate species and subspecies clusters. The same color denotes the same species cluster.

A phylogenetic tree was constructed from the 16S rRNA sequences of the complete genomes of *Bosea* strains as shown in Figure 3A. Similarly, a phylogenetic tree based on different housekeeping genes such as *rpoB*, *gyrB*, *atpD*, *dnaK*, *recA*, and *trpB* is shown in Supplementary Figure S1. Phylogenetic studies using ANI values reflect more effectively the functional relationships involving strains as compared to 16S rRNA sequence studies (Chung et al., 2018).

### 3.3 Genome-based taxonomic analysis by TYGS and ANI

**Figure 4:**
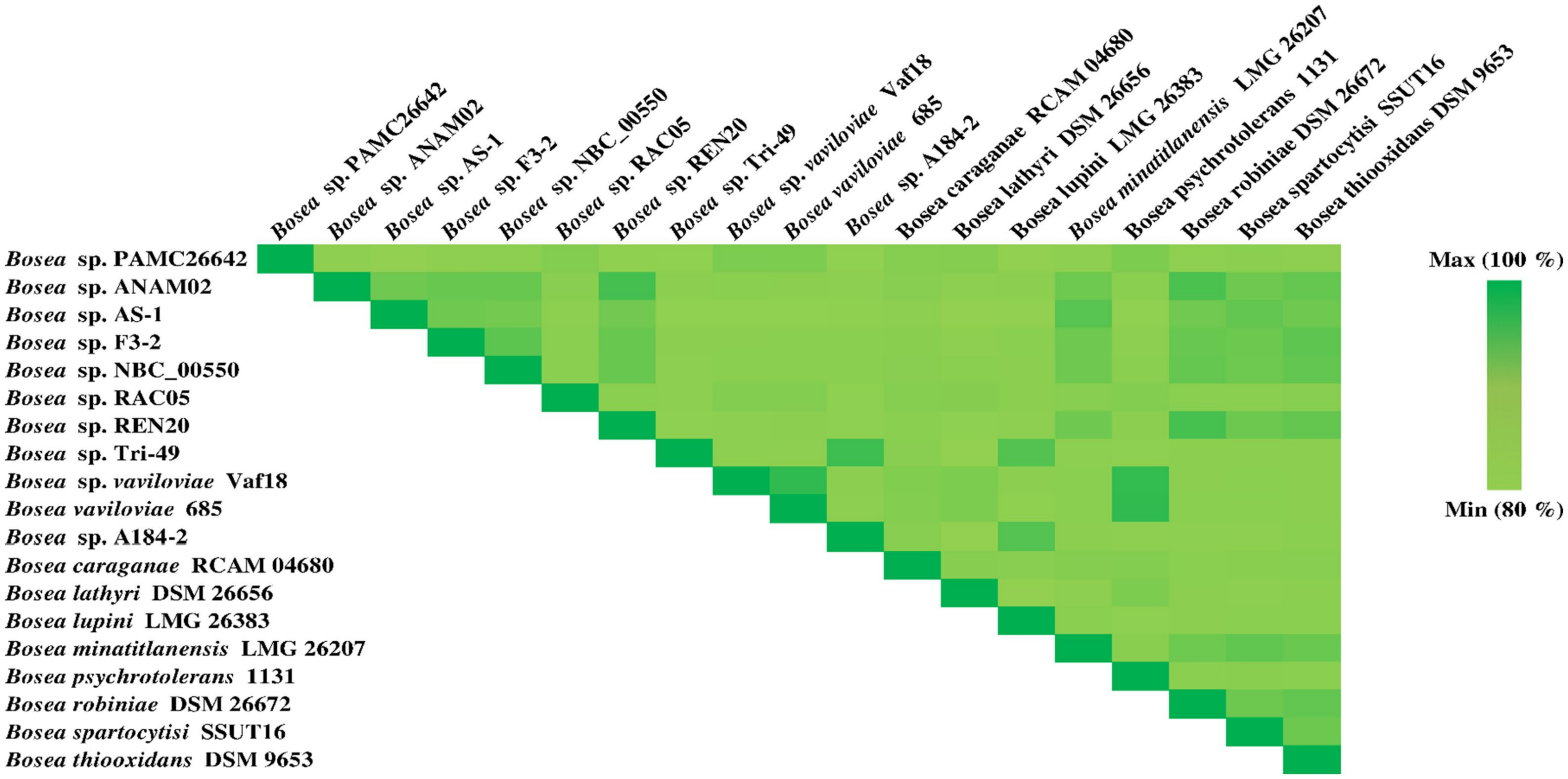
Heatmap generated using the OrthoANI values calculated from OAT software for *Bosea* sp. PAMC26642 with other closely related *Bosea* sp. including the type strain.

Genome Blast Distance Phylogeny (GBDP) analysis was calculated using the TYGS. Phylogenomic tree was shown in Figure 3B. TYGS showed that the ANI value obtained with the complete genome of *Bosea* sp. PAMC26642 is lower than the atypical 95–96% ANI value than the other closely related strain (Figure 4). This confirms that the *Bosea* sp. PAMC26642 might be a potential new species (Meier-Kolthoff and Göker, 2019; Goris et al., 2007; Richter and Rosselló-Móra, 2009; Meier-Kolthoff et al., 2013). In general, bacterial comparative genome analysis uses the ANI methods. As shown in Figure 4, each ANI value ranged from 79.26 to 100% between the bacteria genomes. Thus, we confirm that comparative genome results are lower than the common ANI values of 92–94. The ANI analysis shows the average nucleotide identity of all bacterial orthologous genes shared between any two genomes. It offers a robust resolution between bacterial strains of the same or closely related species (i.e., species showing 80–100% ANI) (Goris et al., 2007). However, ANI values do not represent genome evolution because orthologous genes can vary widely between the compared genomes. Nevertheless, ANI closely reflects the traditional microbiological concept of DNA–DNA hybridization relatedness for defining species, so many researchers use this method as it considers the fluid nature of the bacterial gene pool and implicitly considers shared functions (Jain et al., 2018). Thus, the ANI value is below the 90% threshold, indicating that the genome of *Bosea* sp. PAMC26642 has diverged significantly and provides insights into their evolutionary history and relationships.

### 3.4 Multiple sequence alignment

The nitrate assimilatory proteins are summarized in Table 3. The protein sequences that were searched against the Nr database to find the top sequences with the most identities for the following multiple sequence alignments showed that nitrate reductase of *Bosea* sp. PAMC26642 showed high identities with *Bosea vaviloviae* (WP_069690485.1) of 86% (Safronova et al., 2015), *Bosea* sp. OK403 (WP_092176404.1) of 85% (Li et al., 2023), and *Bosea lathyri* (WP_244595725.1) of 85%. Similarly, for nitrite reductase, our strain showed the highest similarities with *Bosea* sp. Tri-44 (WP_129155718.1) of 91%, *Bosea lathyri* (WP_103874740.1) of 91%, and *Bosea* sp. Root483D1 (WP_057188657.1) of 88% (Karimi et al., 2020). For glutamine synthetase, our strain showed similarities of 98% with *Bosea* sp. 124 (WP_108049945.1), 98% with *Bosea* sp. *lathyri* (WP_103871513.1), and 97% with (WP_054207853.1), for glutamate synthase 93% similarities with *Bosea* sp. AAP35 (WP_197279720.1), 92% with *Bosea* sp. R86505 (WP_376984921), and 77% with *Bradyrhizobium* sp. LTSPM299 (WP_245322060.1), for glutamate dehydrogenase 88% similarities with *Bosea vaviloviae* (WP_069689058.1), 88% similarities with *Bosea psychrotolerans* (WP_103721262.1) (Albert et al., 2019), and 87% similarities with *Bosea lathyri* (WP_200828109.1) (De Meyer and Willems, 2012).

**Table 3:** Multiple sequence alignments of nitrate assimilation proteins.

Proteins	Significant alignments (accession number)	Alignment identifies (%)
Nitrate reductase	*Bosea vaviloviae* (WP_069690485.1)	775/902 (86)
	*Bosea* sp. OK403 (WP_092176404.1)	769/902 (85)
	*Bosea lathyri* (WP_244595725.1)	763/902 (85)
Nitrite reductase	*Bosea* sp. Tri-44 (WP_129155718.1)	542/598 (91)
	*Bosea lathyri* (WP_103874740.1)	546/598 (91)
	*Bosea* sp. Root483D1 (WP_057188657.1)	529/598 (88)
Glutamine synthetase	*Bosea* sp. 124 (WP_108049945.1)	459/469 (98)
	*Bosea* sp. *lathyri* (WP_103871513.1)	458/469 (98)
	*Bosea vaviloviae* (WP_054207853.1)	457/469 (97)
Glutamate synthase	*Bosea* sp. AAP35 (WP_054142013.1)	504/540 (93)
	*Bosea* sp. R86505 (WP_376984921)	499/540 (92)
	*Bradyrhizobium* sp. LTSPM299 (WP_245322060.1)	414/539 (77)
Glutamate dehydrogenase	*Bosea vaviloviae* (WP_069689058.1)	1423/1615 (88)
	*Bosea psychrotolerans* (WP_103721262.1)	1415/1615 (88)
	*Bosea lathyri* (WP_200828109.1)	1407/1615 (87)

Furthermore, the protein sequences (nitrate reductase, nitrite reductase, glutamate synthase, glutamine synthetase, and glutamate dehydrogenase) were searched against the Swiss-Prot database to find the top sequences with the most identities are summarized in Supplementary Table S1. Multiple sequence alignment and InterProScan (software package that allows sequences to be scanned against InterPro’s member database signature) of glutamate dehydrogenase protein revealed the presence of ACT1 domain, ACT2 domain, ACT3, and catalytic domain found in all five bacteria (*Bosea* sp. PAMC26642, *Mycolicibacterium smegmatis* MC2 155, *Halomonas elongata* DSM 2581, *Mycobacterium tuberculosis* H37Rv, and *Pseudomonas aeruginosa* PAO1) compared, including *Bosea* sp. PAMC26642. The ACT domains have been reported to play a significant role in allosteric regulation and structural stability. The catalytic domain is involved in the activity characteristics of GDH, i.e., the reversible oxidative deamination of glutamate to *α*-ketoglutarate and ammonia, which is a central step in nitrogen metabolism (Lázaro et al., 2021; Sharkey et al., 2013; Kawakami et al., 2007; Plaitakis et al., 2017) (see Supplementary Tables S2.1, S5.1 for more detail). The presence of these domains in all bacteria analyzed implies these domains are essential for GDH functionality and likely share similar metabolic strategies. Similarly, a comparison of multiple sequence alignment of glutamate synthase protein in all five bacteria (*Bosea* sp. PAMC26642, *Pyrococcus furiosus* DSM 3638, *Geobacter sulfurreducens* KN400, *Escherichia coli* K-12, *Halomonas elongata* DSM 2581) revealed the presence 4Fe-4S ferredoxin-type iron–sulfur binding domain in *Bosea* sp. PAMC26642 and *Escherichia coli* K-12. This domain facilitates the transfer of electrons from reduced ferredoxin or NADPH to the enzyme active site, where the reductive conversion of glutamine-derived ammonia and 2-oxoglutarate into two molecules of glutamate occurs. The NADPH binding motif (conserved GXGXXG sequence) was identified in the glutamate synthase protein of all five compared bacteria including our strain. However, one amino acid was different in our strain. The details are summarized in Supplementary Table S2.2 and Supplementary Figure S5.2. The NADPH binding motif is important for protein function (Vanoni and Curti, 1999; Vanoni and Curti, 2008; Morandi et al., 2000).

Multiple sequence alignment and InterProScan of glutamine synthetase protein revealed the presence of glutamine synthetase (GS) beta-grasp domain and catalytic domain (see Supplementary Table S2.3; Supplementary Figure S5.1.3 for more detail), which are present in all compared bacteria (*Bosea* sp. PAMC26642, *Bradyrhizobium diazoefficiens* USDA 110, *Azorhizobium caulinodans* ORS 571, *Sinorhizobium meliloti* 1,021, *Rhizobium leguminosarum* bv*. Viciae*). The beta-grasp domain of glutamine synthetase plays an important role in protein stability, functionality, and protein–protein interactions (Burroughs et al., 2007). The preservation of the beta-grasp of glutamine synthetase domain among the bacteria including *Bosea* sp. PAMC26642 indicates they are evolutionarily conserved to maintain their functionality. The catalytic domain performs the enzyme core function. It catalyzes the ATP-dependent synthesis of glutamine from glutamate and ammonium ions.

Multiple sequence alignment and InterProScan of nitrate reductase protein of five bacteria compared (*Bosea* sp. PAMC26642, *Klebsiella oxytoca*, O33732.2, *Shewanella frigidimarina* NCIMB 400, *Synechococcus elongatus PCC 7942 = FACHB-805*, *Synechocystis* sp. PCC 6803 *substr. Kazusa*) reveals the presence of MopB-Nitrate-R-NapA-like domain in all compared bacteria. This domain is reported to be found typically in components of the bacterial nitrate reductase (Nap) complex, especially in the Nap A subunit, which is crucial for nitrate reduction. MopB-Nitrate-R-NapA-like domain facilitates interactions between MoCo and other cofactors, ensuring efficient electron flow to reduce nitrate. MopB-CT was identified in all except *Klebsiella oxytoca*, O33732.2. MopB-CT-Nitrate-R-NapA-like domain stabilizes the structure of the catalytic subunit. In addition, the Molybdop-Fe4S4–2 domain was also identified in all compared bacteria. The core function of this domain has been reported to transfer electrons and stabilize the incorporation of the 4Fe-4S cluster and molybdenum cofactor into the protein structure, ensuring proper enzyme functionality (Sparacino-Watkins et al., 2014; Coelho and Romao, 2015; Moreno-Vivián et al., 1999). The details of domain comparisons are summarized in Supplementary Table S2.4 and Supplementary Figure S5.4. Multiple sequence alignment and InterProScan of nitrite reductase protein reveal the presence of nitrite/sulfite reductase ferredoxin-like half domain present in all five compared bacteria (*Bosea* sp. PAMC26642, *Synechococcus elongatus* PCC 7942, *Leptolyngbya laminosa, Mycobacterium tuberculosis* CDC1551 and *Mycobacterium avium* subsp. paratuberculosis K-10). The details regarding the comparison of domains are summarized in Supplementary Table S2.5 and Supplementary Figure S5.5.

### 3.5 Nitrogen metabolism, KEGG pathway, and putative 3D structure modeling

**Figure 5:**
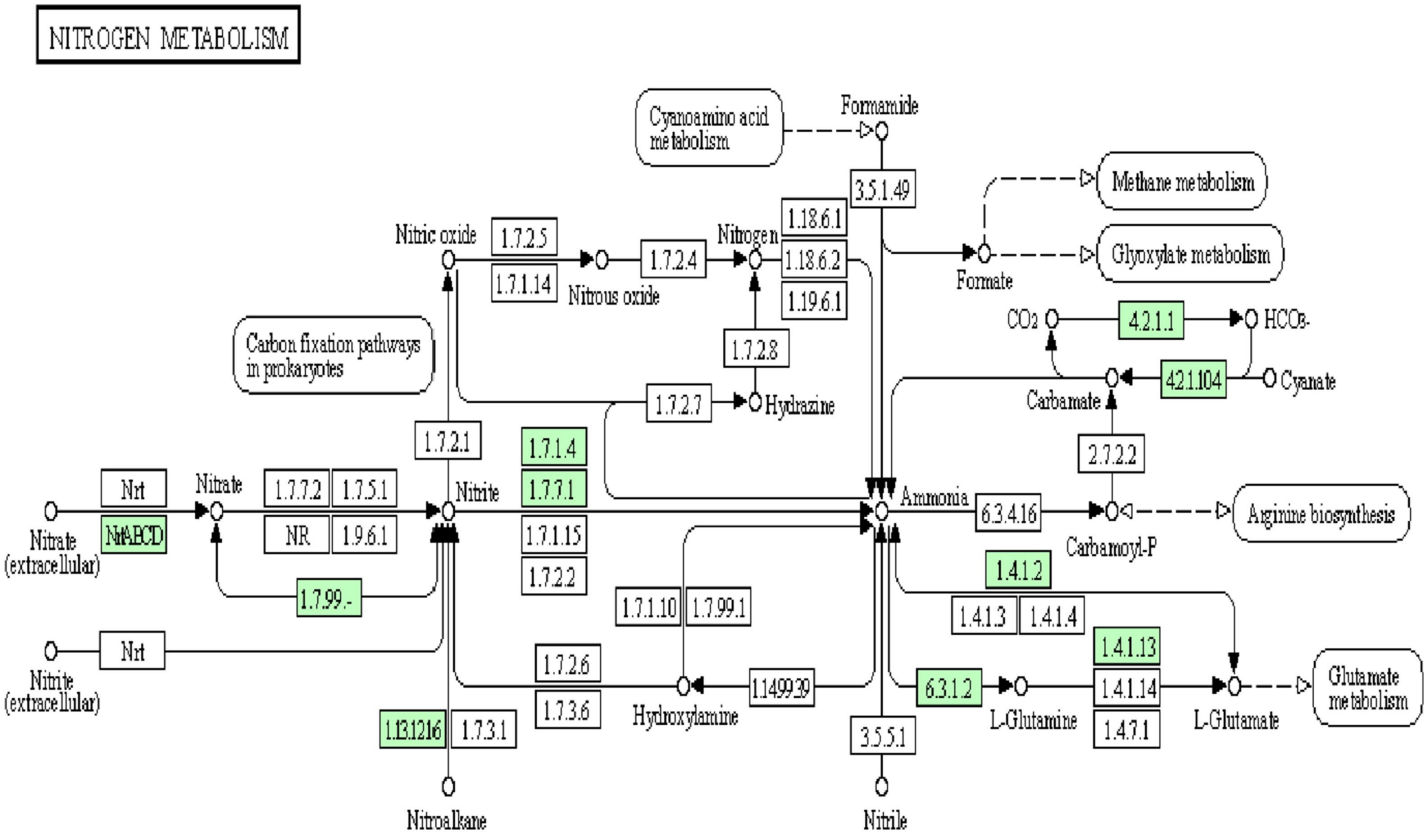
Snapshot of KEGG database showing nitrogen metabolism pathway in *Bosea* sp. PAMC26642. The enzymes available are highlighted in green.

The genome analysis of *Bosea* sp. PAMC26642 showed the presence of nitrogen metabolic enzymes, transcription factors, and transporters. Among the three different nitrate-reducing systems (Nas, Nar, and Nap) reported in prokaryotes (Moreno-Vivián et al., 1999; González et al., 2006), the assimilatory pathway was identified in *Bosea* sp. PAMC26642. Nitrate reductase (EC 1.7.99.4), NAD(P)H-nitrite reductase (EC 1.7.1.4), ferrodoxin-nitrite reductase (EC 1.7.7.1), glutamine synthetase (EC 6.3.1.2), glutamate synthase (EC 1.4.1.13), and glutamate dehydrogenase (EC 1.4.1.2), which catalyzes the reversible conversion between 2-oxoglutarate/ammonium and glutamate using NAD(H) or NADP(H) as a coenzyme (Smith et al., 1975) was also identified. In addition to that, cyanate lyase (EC 4.2.1.104), an enzyme responsible for catalyzing the decomposition of cyanate in a bicarbonate-dependent reaction yielding carbamate, which spontaneously decarboxylates to ammonia and carbon dioxide (Johnson and Anderson, 1987), was also identified. Furthermore, carbonic anhydrase (EC 4.2.1.1) and nitronate monooxygenase (EC 1.13.12.16) were also identified. In addition, nitrate/nitrite transporter substrate-binding protein (Figure 5) was also identified. Nitrate is transported into a cell by an active transport system. Nitrate is converted to nitrite with the function of nitrate reductase, followed by the reduction in nitrite to ammonia and then the conversion of ammonia to glutamine through nitrite reductase and glutamine synthetase. Finally, glutamine is transformed into glutamate by glutamate synthase. Both glutamine and glutamate are essential substrates for protein synthesis and energy metabolism. Glutamate is metabolized into ammonia and *α*-ketoglutarate with glutamate dehydrogenase.

**Figure 6:**
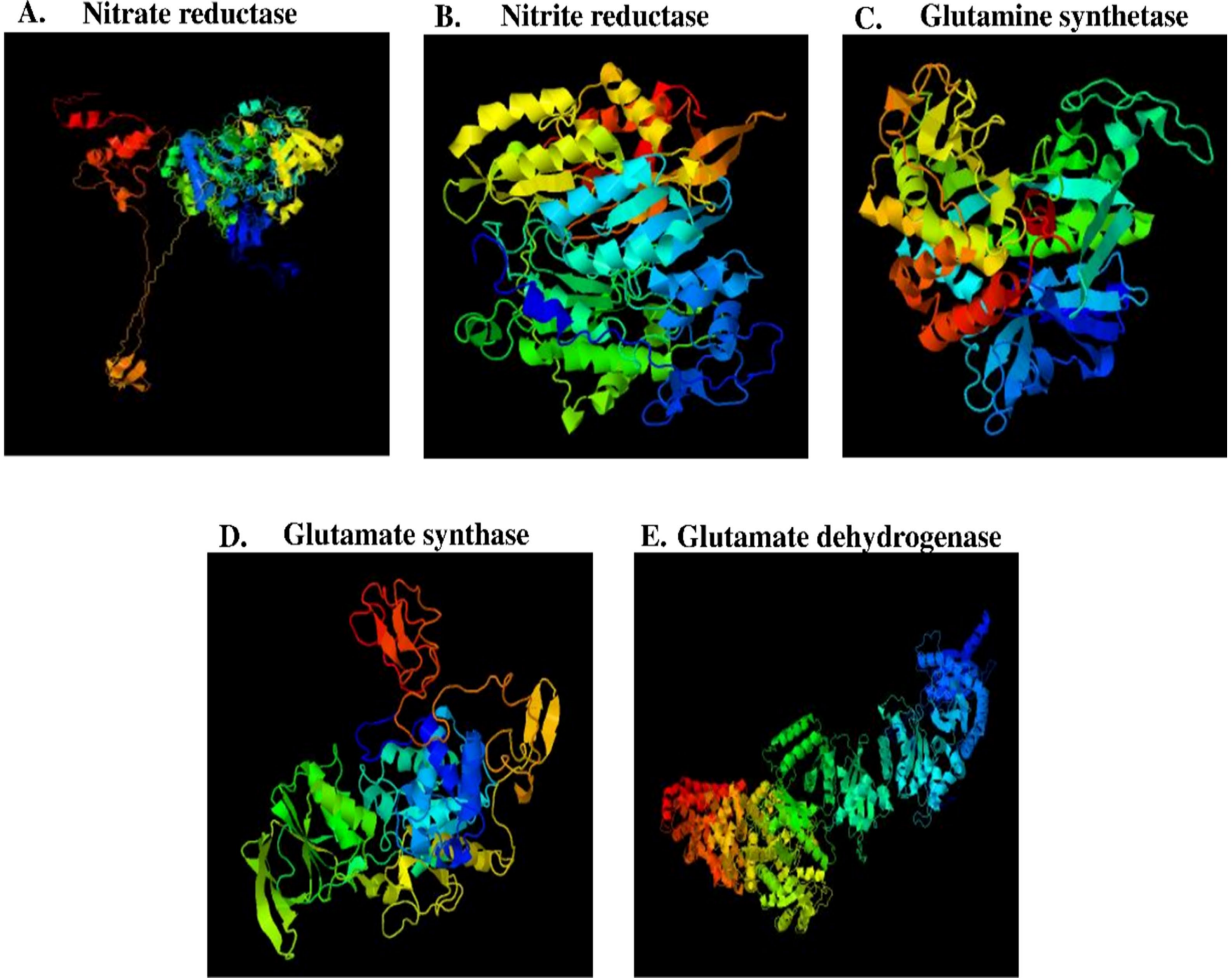
Putative 3D model of nitrate assimilatory proteins. **(A)** Nitrate reductase protein **(B)** Nitrite reductase protein **(C)** Glutamine synthetase protein, and **(D)** Glutamate synthase **(E)** Glutamate dehydrogenase protein.

A putative 3D model for all the nitrate assimilatory pathway proteins (nitrate reductase, nitrite reductase, glutamine synthetase, glutamate synthase, and glutamate dehydrogenase) is shown in Figure 6, and its information is shown in Table 4. Putative 3D structure modeling of (1) nitrate reductase was generated based on the sequence residues from 13–716 with 77% coverage, 100% confidence, and 35% identity against C2v45A (2.80 Å). (2) nitrite reductase was generated based on the sequence residues from 51 to 588 with 89% coverage, 100% confidence, and 30% identity against C1Zj8B (2.80 Å). (3) glutamine synthetase was generated based on the sequence residues from 3 to 468 with 99% coverage, 100% confidence, and 62% identity against C1fpyE (2.89 Å). (4) glutamate synthase was generated based on the sequence residues from 15 to 534 with 96% coverage, 100% confidence, and 31% identity against C1gthD (2.25 Å). (5) glutamate dehydrogenase was generated based on the sequence residues from 39 to 1,616 with 97% coverage, 100% confidence, and 39% identity against C7jsrA (6.27 Å).

**Table 4:** Putative 3D modeling information of nitrate assimilatory proteins.

Proteins	Residue range (AA)	Query coverage (%)	Identity (%)	Confidence (%)	Template (Å)
Nitrate reductase	13–716	77	35	100	C2v45A (2.40)
Nitrite reductase	51–588	89	30	100	C1Zj8B (2.80)
Glutamine synthetase	3–468	99	62	100	C1fpyE (2.80)
Glutamate synthase	15–534	96	31	100	C1gthD (2.25)
Glutamate dehydrogenase	39–1,616	97	39	100	C7jsrA (6.27)

### 3.6 Nitrate reduction assay (qualitative measurement) at different temperatures

**Figure 7:**
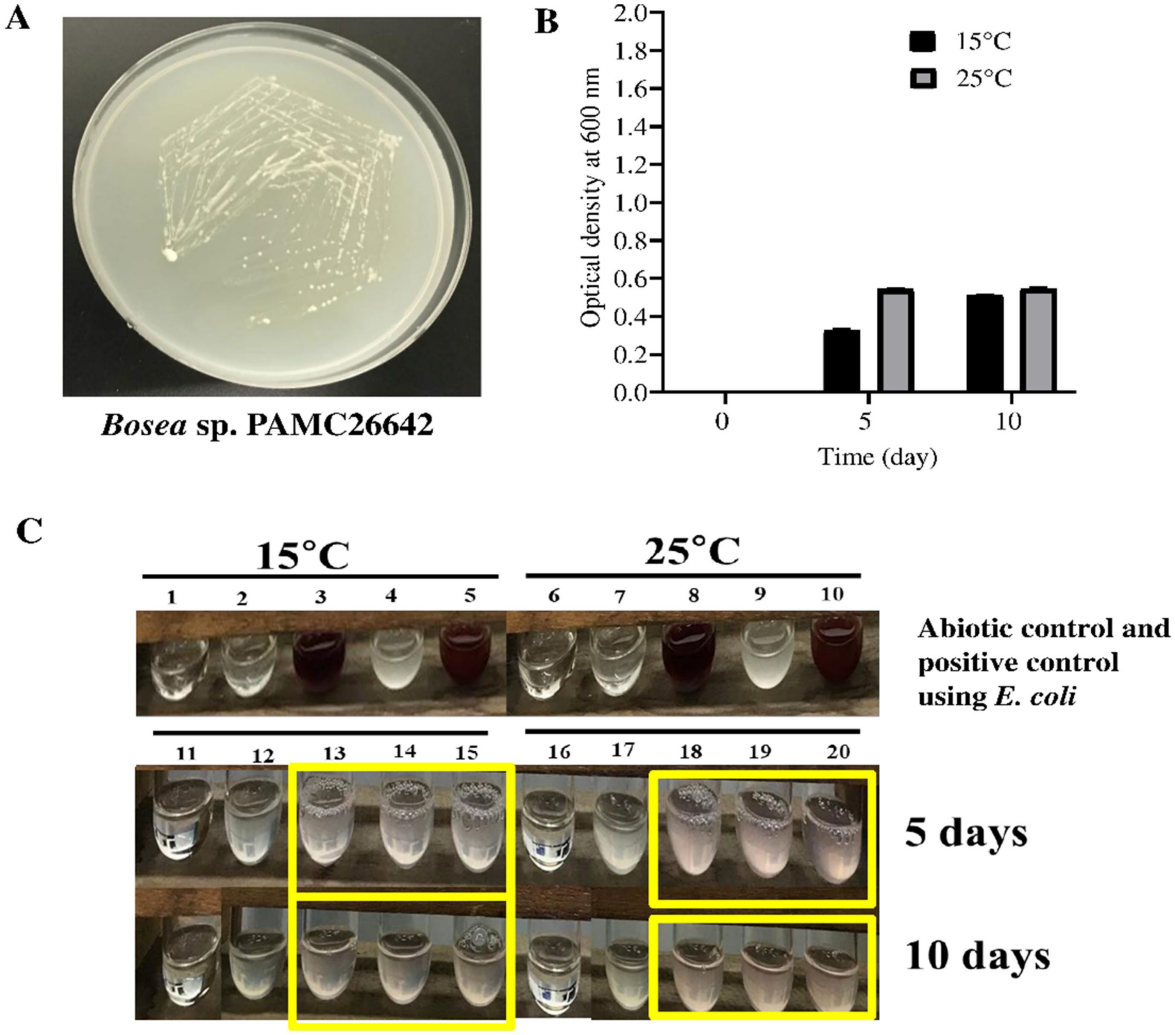
**(A)** *Bosea* sp. PAMC26642 in R2A media. **(B)** Growth of bacteria in nitrate media at different times and at different temperatures. The black box represents nitrate media containing cells at 15°C, and grey box represents nitrate media containing cells at 25°C. **(C)** Nitrate reduction assay at 15°C and 25°C. 1 and 6, abiotic control at 15°C and 25°C; 2 and 7, abiotic control with reagent A and reagent B at 15°C and 25°C; 3 and 8, abiotic control with reagent A, reagent B, and addition of zinc at 15°C and 25°C; 4 and 9, *E. coli* without reagent A and reagent B at 15°C and 25°C; 5 and 10, positive control using *E. coli* with reagent A and reagent B at 15°C and 25°C; 11 and 16, abiotic control at 15°C and 25°C; 12 and 17, *Bosea* culture without reagent A and reagent B; and 13, 14, 15, 18, 19, and 20, *Bosea* culture with reagent A and reagent B at 15°C and 25°C, respectively. Nitrate assays were performed at days 5 and 10. Experiments were performed in triplicates.

The nitrate reduction assay was performed, which is based on the ability of bacteria to reduce nitrate to nitrite with the liberation of red color from a colorless solution. *Bosea* sp. PAMC26642 was tested for the nitrate reduction assay, including an abiotic control (without microorganisms) and *E. coli* as a positive control at temperatures of 15°C and 25°C. Both *Bosea* sp. PAMC26642 and *E. coli* showed reductions of nitrate at 15°C and 25°C. However, an abiotic control did not show any color change (Figure 7). The ability of bacteria to reduce nitrate at specific temperatures is a key adaptive trait, crucial for their survival across various environments. Furthermore, the enzymes required for nitrate reduction, primarily nitrate reductase, have temperature-dependent activity. These enzymes either work inefficiently or cease to function at temperatures outside their optimal range. The bacteria able to reduce nitrate at certain temperatures have implications such as ecological niche specialization, which minimizes the competition with other microorganisms. In addition to that, they also influence nutrient availability, overall ecosystem balance, survival, and adaptation to climate change. The result of nitrate reduction by *Bosea* sp. PAMC26642 at different temperatures (15°C and 25°C) revealed that the activity of nitrate reductase is functioning appropriately.

This distinguishing feature of *Bosea* sp. PAMC26642 (in this study) is that they have a nitrogen assimilation pathway (nitrogen cycling) and its associated enzymes, which is important for adaptation in cold areas because nitrogen availability is a limiting factor in polar regions of the Arctic due to cold temperatures and nutrient-poor environment.

### 3.7 Proteins to be involved in different stress adaptation mechanisms

Aside from nitrogen metabolic enzymes, other enzymes involved in various stress adaptation mechanisms such as oxidative stress (superoxide dismutase, catalase, and thiol peroxidase), heavy metal resistance (arsenate reductase, ArsH), temperature stress (Csp and Hsp), and osmotic stress (OmpR) were identified in *Bosea* sp. PAMC26642. The comparison of this strain with other strains of the same genus in terms of genes predicted to be involved in different stress adaptation mechanisms after the annotation with the RAST server is shown in Table 5.

Among different types of stress temperature stress, either low or high temperature, bacteria have been reported to have evolved several mechanisms for coping with temperature stress and adapting to changing environmental conditions such as the production of cold shock protein (Csp) during low temperature. In addition, these Csps have been reported to contribute to osmotic, oxidative, starvation, pH, and ethanol stress tolerance (Keto-Timonen et al., 2016). In addition to Csps, there are other heat shock proteins (HSP) that are reported to be involved during bacterial high temperature-related environmental stress. HSP is found in various types of bacteria (Maleki et al., 2016), major Hsps are molecular chaperons GroEl-GroES, DNAJ, and GrpE, which were reported for regulation of folding as well as for heat shock response such as in *E. coli* (Nishihara et al., 1998; Arsène et al., 2000) and were identified in all the *Bosea* strains including HtpX (Kornitzer et al., 1991), whereas Hsp20 (Bakthisaran et al., 2015) was identified only in some *Bosea* strain. Furthermore, sigma factors play a crucial role in bacterial adaptability and survival, because of these factors the bacteria can swiftly change their gene expression profiles in response to environmental signals, optimizing their metabolic, growth, and stress responses. In addition, multiple sigma factors in bacteria have been reported to provide a mechanism for global coordinate regulation of classes of genes (Burgess, 2013). Different types of sigma factors such as RpoN, RpoE, RpoH, and RpoD have been reported in bacteria such as *E. coli* K-12, *Pseudomonas aeruginosa*, and *Shewanella oneidensis* MR-1 (Shimada et al., 2021; Potvin et al., 2008; Dai et al., 2015). Among these sigma factors, RpoD, RpoH, and RpoN were identified in all *Bosea* strains (Table 5). Osmotic stress-related protein, EnvZ/OmpR two-component system, which mediates osmotic stress response in several Gram-negative bacteria (Yuan et al., 2011), and EnvZ was identified in all *Bosea* strains. Superoxide dismutase (SOD), which converts superoxide radicals to the less toxic H_2_O_2_ and water. They were reported to be varied in microbes such as cytoplasmic Mn-SOD (encoded by *sodA*), Fe-SOD (encoded by *sodB*), and periplasmic Cu/Zn-SOD (encoded by *sodC*) (Seib et al., 2006; Sheehan et al., 2000; Najmuldeen et al., 2019). Among these three SODs, Mn-SOD was identified in all the *Bosea* strains and Cu/Zn-SOD was not identified in any of the *Bosea* strains including our strain. Catalases known for their protection against H_2_O_2_ (Katsuwon and Anderson, 1989) are classified into three groups, monofunctional heme-containing catalases (KatE), heme-containing catalase-peroxidase (KatG), and manganese-containing catalases. Among these catalases, KatE were identified in *Bosea* sp. PAMC26642, *Bosea* sp. NBC_00550, and *Bosea* sp. REN20. Manganese-catalase was identified in *Bosea* sp. NBC_00550 and *Bosea vestrisii*.

**Table 5:** Comparison of proteins that are involved in different stress adaptation mechanisms of all the *Bosea* strains having a complete genome.

	Strain name											Functions
	*Bosea* sp. PAMC 26642	*Bosea* sp. ANAM02	*Bosea* sp. 685	*Bosea* sp. AS-1	*Bosea* sp. F3-2	*Bosea* sp. NBC_00550	*Bosea* sp. RAC05	*Bosea* sp. REN20	*Bosea* sp. Tri-49	*Bosea vaviloviae*	*Bosea vestrisii*	
Type of stress												
1. Temperature stress												
Type of Protein												
RNA polymerase sigma factor (RpoD)	+	+	+	+	+	+	+	+	+	+	+	
RNA polymerase sigma factor (RpoH)	+	+	+	+	+	+	+	+	+	+	+	
RNA polymerase sigma-54 factor (RpoN)	+	+	+	+	+	+	+	+	+	+	+	
RNA polymerase sigma factor (RpoE)	−	*	−	−	*	*	*	*	*	*	*	Regulates the degQ and supports growth at low and high temp
RNA polymerase sigma-70 factor	*	+	+	*	−	−	−	+	+	+	+	
Cold shock protein of Csp family	+	+	+	+	+	+	+	+	+	+	+	Protects the bacteria during rapid downshift of temperature
Hsp family (GrpE: GroES, GroEL)	+	+	+	+	+	+	+	+	+	+	+	
Heat shock protein DnaJ-like	−	+	+	+	+	+	+	*	+	+	+	Involved in protein folding and refolding, expressed in high temperature
Heat shock protein (Hsp20)	*	−	*	+	*	−	−	−	−	−	−	
Heat shock protein (HtpX)	+	+	+	+	+	+	+	+	+	+	+	
2. Osmotic stress												Mediates osmotic stress response in a number of Gram-negative bacteria
Osmolarity sensor protein (EnvZ)	+	+	+	+	+	+	+	+	+	+	+	
Two-component transcriptional response regulator (OmpR)	+	+	+	+	+	+	+	+	−	+	−	
3. Oxidative stress												
i. superoxide dismutase												Provides superoxide resistance
(Mn superoxide dismutase)	+	+	+	+	+	+	+	+	+	+	+	
(Fe superoxide dismutase)	−	−	−	−	−	−	+	−	−	−	−	
(Cu/Zn superoxide dismutase)	−	−	−	−	−	−	−	−	−	−	−	
ii. Catalase												Protection against the H_2_O_2_
KatE	+	−	−	−	−	+	−	+	−	−	−	
Manganese catalase	+	−	−	−	−	+	−	−	−	−	+	
KatG	*	+	+	+	+	−	+	+	+	+	+	
iii. Hydroperoxide reductase												
Organic hydroperoxide resistance protein	+	+	+	+	+	+	+	+	+	+	+	Oxidative stress defense
Organic hydroperoxide resistance transcriptional regulator	+	+	+	+	+	+	+	+	+	+	+	
iv. Alkyl hydroperoxide reductases												
Alkyl hydroperoxide reductase (AHP1)	*	*	−	−	−	−	−	−	−	−	−	
Alkyl hydroperoxide reductase protein C (AhpC)	*	*	−	−	−	+	+	+	+	−	+	
Alkyl hydroperoxidase AhpD family core domain protein	*	+	+	+	+	+	−	+	+	+	+	
Alkyl hydroperoxide reductase subunit C-like protein	*	*	−	−	+	−	−	+	−	−	−	
v. Thiol peroxidase												Reduces t-butyl hydroperoxidase, H_2_O_2_, and cumene hydroperoxidase
Thiol peroxidase Tpx-type	−	−	−	−	−	−	−	−	−	−	−	
Thiol peroxidase Bcp type	+	+	+	+	+	+	+	+	+	+	+	
4. HMs resistance												
Chromate transport protein (ChrA)	+	+	+	+	+	+	+	+	+	+	+	Provides resistance to chromate
Magnesium and cobalt transport protein (CorA)	+	+	+	+	+	+	+	+	+	+	+	Provides resistance to cobalt-zinc-cadmium
Magnesium and cobalt efflux protein (CorC)	+	+	+	+	+	+	+	+	+	+	+	Transport magnesium and cobalt
Nickel/cobalt efflux transporter (RcnA)	+	−	+	−	−	−	−	−	−	+	+	
Cobalt-zinc-cadmium resistance protein	+	+	+	+	+	+	+	+	+	+	+	
Cobalt transporter (CbtA)	+	−	−	+	*	−	−	−	−	+	−	
Cobalt transporter (CbtB)	−	−	−	−	−	−	−	−	−	−	−	
Cobalt ABC transporter, ATP-binding protein (CbtL)	−	+	−	−	+	+	−	+	+	−	+	
Cobalt ABC transporter, permease protein (CbtK)	−	+	−	−	+	+	−	+	+	−	+	
Cobalt ABC transporter, substrate-binding protein (CbtJ)	−	+	−	−	+	+	−	+	+	−	+	
Arsenate reductase	+	+	+	+	+	+	+	+	+	+	+	Provides resistance to arsenic
Arsenical-resistance protein (Acr3)	−	+	+	+	+	+	−	+	+	+	+	Provides resistance to arsenic
Arsenite/antimonite: H+ antiporter (ArsB)	−	−	−	−	−	−	+	−	−	−	−	
Flavin-dependent monooxygenase ArsO associated with arsenic resistance	−	−	−	−	−	−	+	−	−	−	−	
Arsenate reductase (ArsH)	+	−	−	+	−	−	−	−	−	+	−	
Transcriptional regulator (ArsR family)	+	+	+	+	+	+	+	+	+	+	+	
Copper tolerance protein	*	+	+	+	+	+	+	+	+	+	+	Provides resistance to copper
Cu(I)-responsive transcriptional regulator	+	+	+	+	+	+	+	*	*	+	*	

“+” Presence of protein. “−” Absence of protein. “*” May be present or absent but not specified.

Organic hydroperoxide resistance protein (Ohr) and organic hydroperoxide resistance regulator (OhrR), which are critical for organic peroxide resistance (Si et al., 2020) and could be involved in the detoxification of organic hydroperoxide (Shea and Mulks, 2002) were identified in all the *Bosea* strains. Peroxiredoxins are widespread in bacteria and are of two types of thiol peroxidase (Tpx), and the bacterioferritin co-migratory protein (Bcp) has been reported to play a role in the protection against oxidative stress, particularly that caused by excess oxygen and exogenous peroxidase as reported in *Campylobacter jejuni* (Atack et al., 2008). Furthermore, Tpx and Bcp appear to be able to use a wide variety of peroxide as substrates *in vitro*, such as hydrogen peroxide, organic peroxides, and lipid peroxides (Cha et al., 2004; Rho et al., 2006; Wang et al., 2005). Thiol peroxidase Bcp type was identified in all the *Bosea* strains. Heavy metal(loid)s toxicity has been considered as a global issue, and they have been considered as a serious environmental problem. Bacteria have been reported to have developed a resistance toward heavy metal(loid)s because of the presence of certain resistance genes. ArsH protein (organo arsenical oxidase) responsible for arsenic biotransformation (Chang et al., 2018) was identified in *Bosea* sp. AS-1, *Bosea* sp. Tri-49, and *Bosea vaviloviae* including our strain.

Similarly, the Acr3 protein belongs to the bile/arsenate/riboflavin transporter (BART) superfamily and is reported to be widespread in bacteria, archaea, fungi, and some plants (Mansour et al., 2007) was identified in all *Bosea* strains except *Bosea* sp. PAMC26642. Chromate transport protein (ChrA), membrane protein, and member of the chromate ion transporter protein (CHR) superfamily that confers resistance to the toxic ion chromate through the energy-dependent chromate efflux from the cytoplasm (Díaz-Pérez et al., 2007), was identified in all *Bosea* strains. RcnA, a nickel and cobalt-resistant protein reported in *E. coli* (Rodrigue et al., 2005), was identified in our strain, *Bosea* sp. 685, *Bosea vaviloviae* and *Bosea vestrisii*. HM-related protein (CorA) as reported by Afordoanyi et al. (2023) formerly known as magnesium and cobalt transport protein for mediating both influx and efflux of Mg^2+^ in *Salmonella typhimurium* and *E. coli* (Smith et al., 1998) were identified in all the *Bosea* strains. In addition, magnesium and cobalt transport protein and arsenate reductase were also identified in all the *Bosea* strains.

## 4 Conclusion

This study provides valuable insights into the nitrogen metabolic potential of lichen-associated *Bosea* sp. PAMC26642 from the polar region. It also sheds light on the strain’s stress adaptation mechanism. In this study, this strain has been compared to other species in the *Bosea* genus using comprehensive bioinformatics tools and wet-lab assays. The key enzymes of the assimilatory nitrogen metabolic pathway such as nitrate reductase, nitrite reductase, glutamine synthetase, glutamate synthase, and glutamate dehydrogenase were identified in *Bosea* sp. PAMC26642. In particular, the strain demonstrated nitrate reduction ability at 15°C and 25°C, highlighting its metabolic adaptability to cold environments. In addition, stress adaptation enzymes suggest resilience to oxidative stress, heavy metal resistance, temperature fluctuations, and osmotic stress. These findings not only expand our understanding of *Bosea* biodiversity and nitrogen metabolic capacity but also highlight its potential applications in ecosystem monitoring, nitrate bioremediation, and environment resilience strategies. This finding lays the foundation for leveraging cold-adapted microorganisms such as *Bosea* sp. PAMC26642 to address environmental challenges and promote ecosystem resilience in a changing climate. Overall, these findings will provide new knowledge gained in key areas like an enhanced understanding of nitrogen cycling in polar ecosystems, insight into cold-adapted metabolic adaptability, stress resilience mechanism in polar microorganisms, potential for bioremediation and environmental application and framework for ecosystem monitoring, and climate adaptation strategy.

## Data Availability

All supporting data and protocols have been provided within the article or through Supplementary files. The datasets analyzed during the current study are available in the NCBI repository, accession numbers: NZ_CP014301.1 for *Bosea* sp. PAMC26642, NZ_ AP022848.1 for *Bosea* sp. ANAM02, NZ_CP042331.1 for *Bosea* sp. F3-2, NZ_CP022372.1 for *Bosea* sp. AS-1, NZ_CP016464.1 for *Bosea* sp. RAC05, NZ_CP017946.1 for Bosea sp. Tri-49, NZ_CP017147.1 for *Bosea vaviloviae* Vaf18, NZ_CP126307.1 for *Bosea vestrisii* A18/4-2, NZ_CP102772.1 for *Bosea* sp. NBC_00550, NZ_ CP134779.1 for *Bosea vaviloviae* 685, and NZ_OR405985.1 for *Bosea* sp. REN20.

## Footnotes

^1^ https://www.ncbi.nlm.nih.gov/

^2^ https://tygs.dsmz.de

^3^ www.ezbiocloud.net/tools/ani

^4^ http://ggdc.dsmz.de/distcalc2.php

^5^ http://espirit.ibcp.fr

^6^ http://genome3d.eu/
