# Supplementary material for: Unraveling nitrogen metabolism, cold and stress adaptation in polar Bosea sp. PAMC26642 through comparative genome analysis
Source: Front Microbiol. 2025 Jan 24;15:1505699. doi: 10.3389/fmicb.2024.1505699 (PMC11804256; doi:10.3389/fmicb.2024.1505699)
Supplement: Supplementary file 1 [file Data_Sheet_1.docx]

*Supplementary*

*materials*

*for*

**Unraveling Nitrogen Metabolism, Cold and Stress Adaptation in Polar *Bosea* sp. PAMC26642 through Comparative Genome Analysis**

Anamika Khanal^1,2^, So-Ra Han^1,2,3^, Jun Hyuck Lee^4^, and Tae-Jin Oh^1,2,3,5*^

^1^Genome-based Bio-IT Convergence Institute, Asan 31460, South Korea; anamika.khanal@gmail.com (A.K.)

^2^Bio Big Data-based Chungnam Smart Clean Research Leader Training Program, SunMoon University, Asan, South Korea; 553sora@hanmail.net (S.-R.H.)

^3^Department of Life Science and Biochemical Engineering, Graduate School, SunMoon University, Asan 31460, South Korea

^4^Research Unit of Cryogenic Novel Materials, Korea Polar Research Institute, Incheon 21990, South Korea; junhyucklee@kopri.re.kr (J.H.L.)

^5^Department of Pharmaceutical Engineering and Biotechnology, SunMoon University, Asan 31460, South Korea

*Corresponding author: Prof. Tae-Jin Oh

Department of Pharmaceutical Engineering and Biotechnology, Sun Moon University, Asan 31460, South Korea. *E-mail address:* tjoh3782@sunmoon.ac.kr (T.-J.O.)

**Running Title:** Comparative genome analysis of bacteria isolated from polar region

**Keywords:** *Bosea* sp. PAMC26642, Cold adaptation, Comparative genomics, Nitrogen metabolism, Gram-negative bacteria.

**Supplementary Figure S1.** Neighbor-joining phylogenetic trees of a housekeeping gene (*dnaK*) without an outgroup using a maximum composite likelihood model. Percentages in the bootstrap test are from 1,000 replicates.


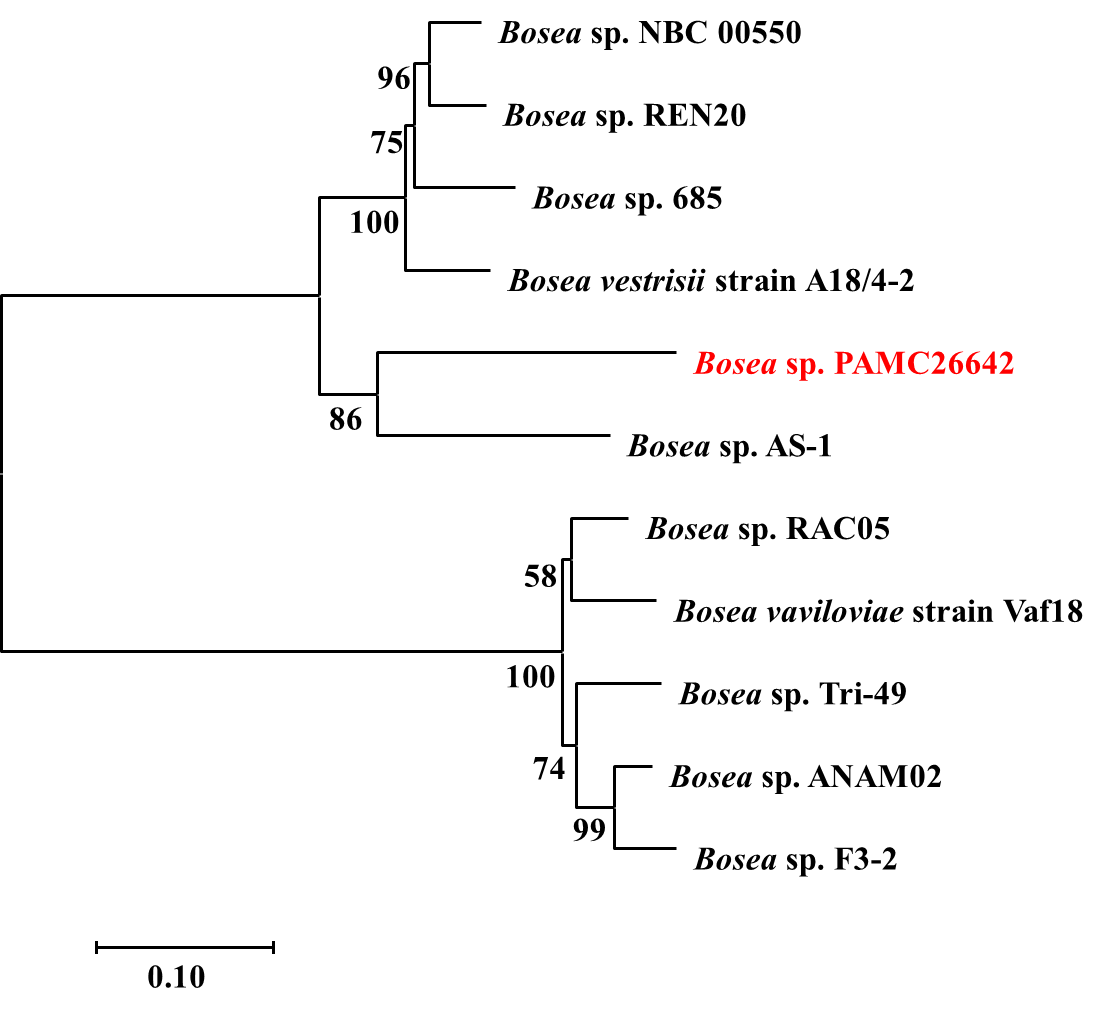


**Supplementary Figure S2.** Neighbor-joining phylogenetic trees of a housekeeping gene (*RecA*) without an outgroup using a maximum composite likelihood model. Percentages in the bootstrap test are from 1,000 replicates.


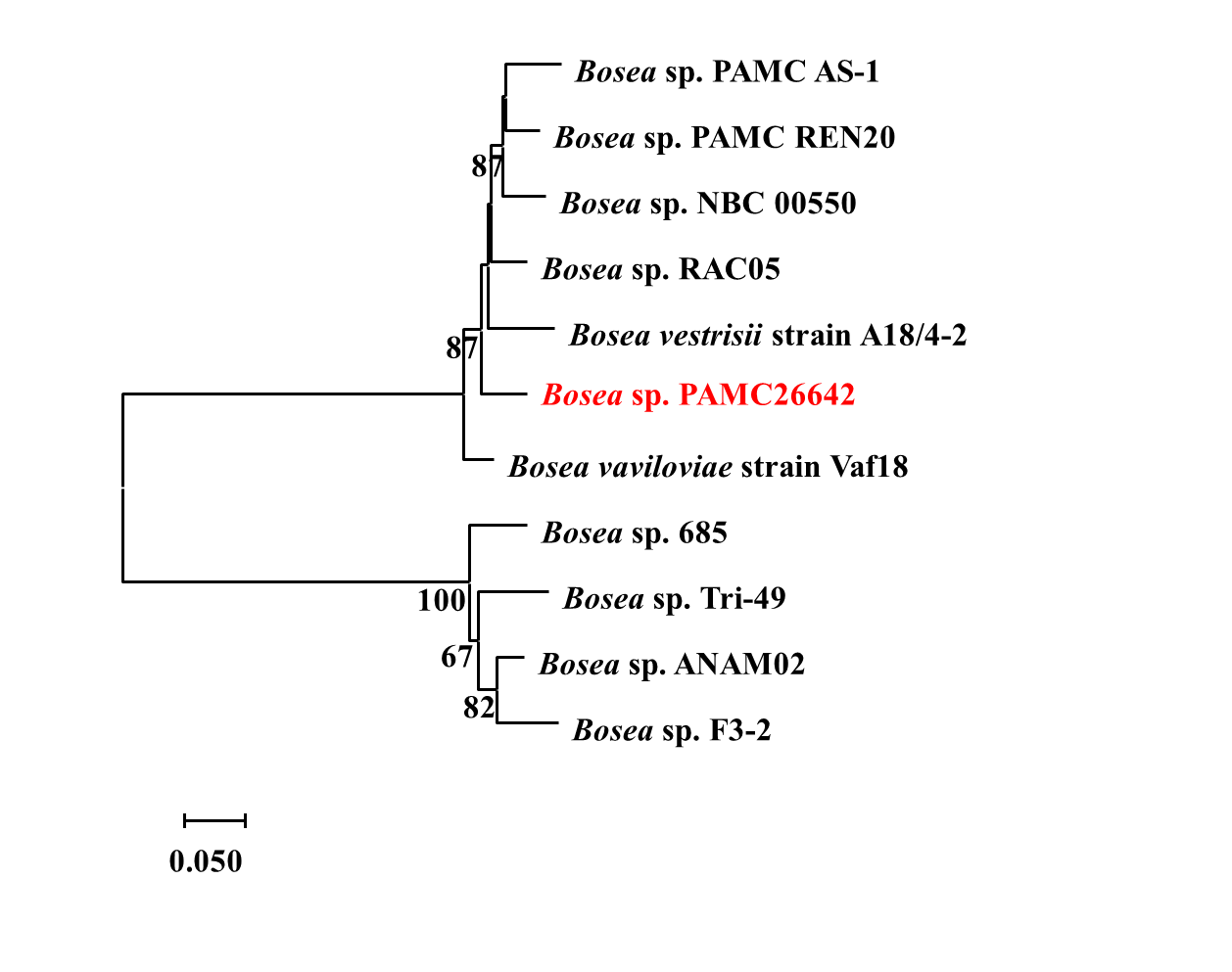


**Supplementary Figure S3.** Neighbor-joining phylogenetic trees of a housekeeping gene (*gyrB*) without an outgroup using a maximum composite likelihood model. Percentages in the bootstrap test are from 1,000 replicates.


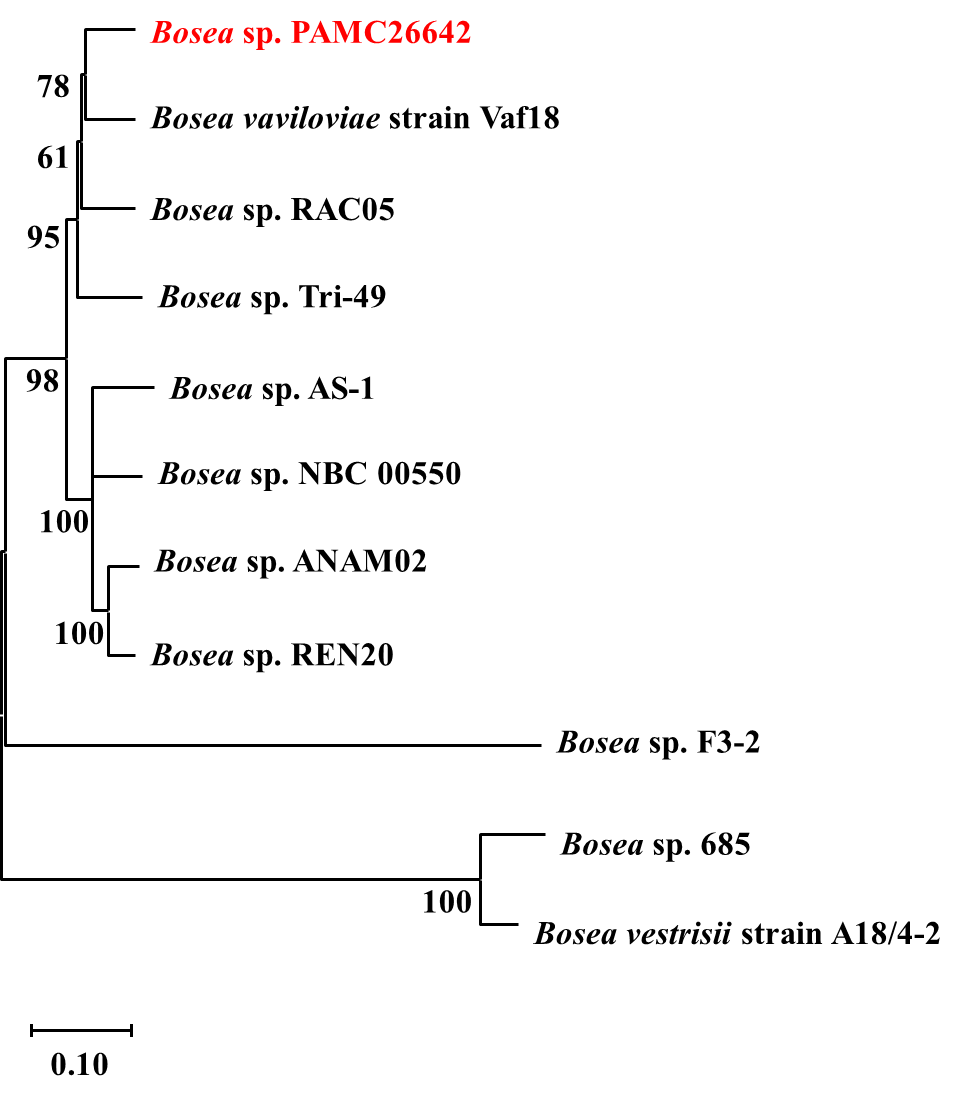


**Supplementary Figure S4.** Neighbor-joining phylogenetic trees of a housekeeping gene (*trpB*) without an outgroup using a maximum composite likelihood model. Percentages in the bootstrap test are from 1,000 replicates.


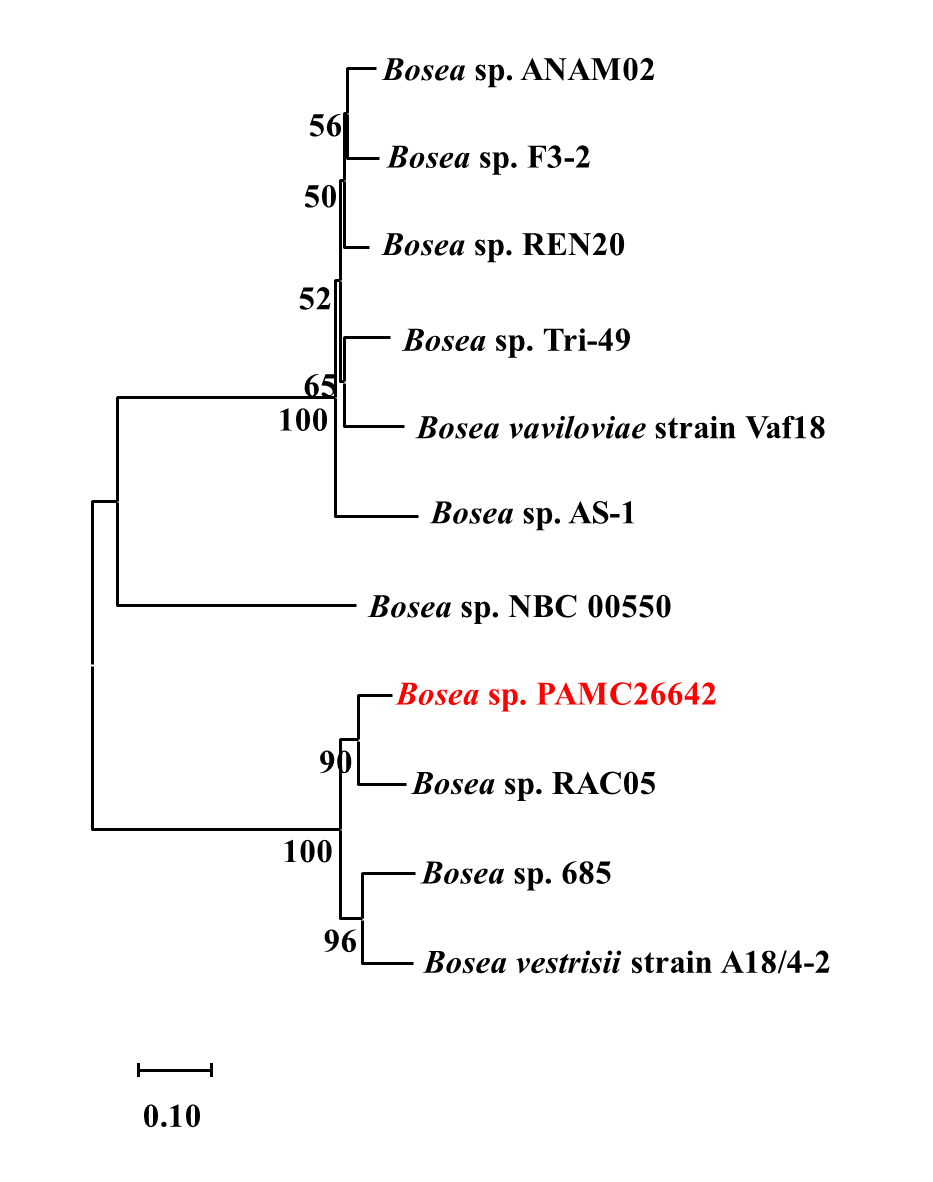


**Supplementary Figure S5.1.** Multiple sequence alignment of glutamate dehydrogenase using Clustal Omega and ESPript 3.0. AMJ62141.1: AMJ62141.1: *Bosea* sp. PAMC26642, E1V4J5.1: *Halomonas elongata* DSM 2581, Q9HZE0.1: *Pseudomonas aeruginosa* PAO1, A0R1C2.1: *Mycolicibacterium smegmatis* MC2 155, O532031: *Mycobacterium tuberculosis* H37Rv.


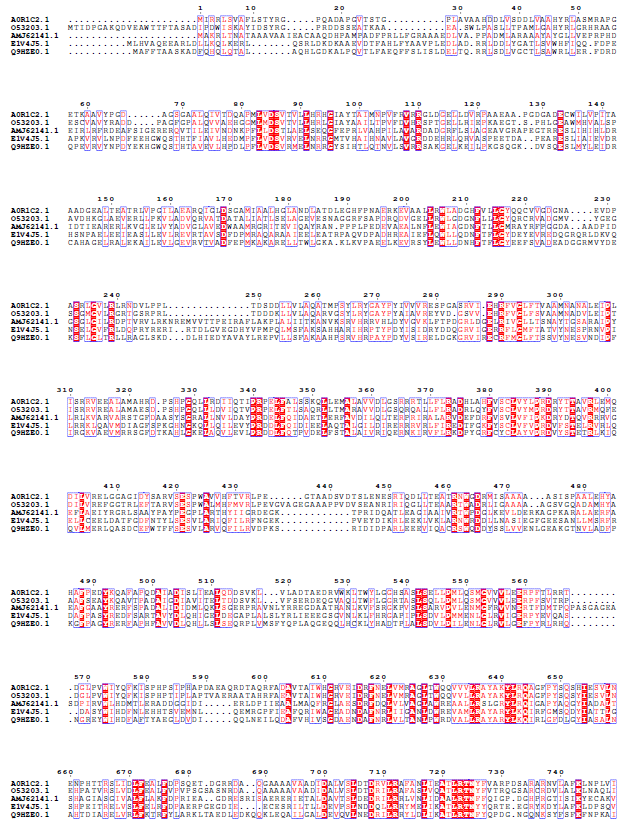


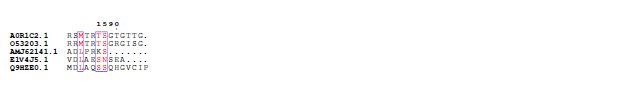

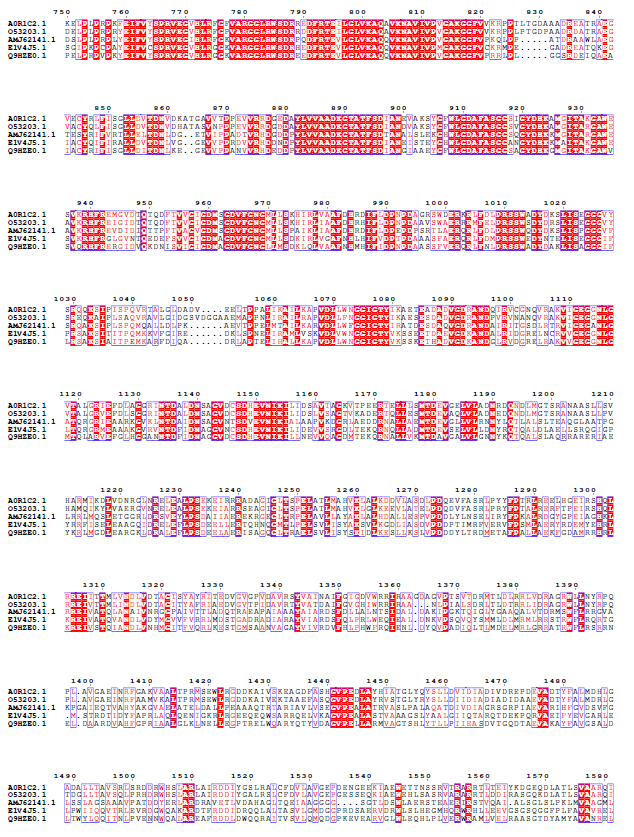


**Supplementary Figure S5.2.** Multiple sequence alignment of glutamate synthase using Clustal Omega and ESPript 3.0. AMJ63452.1: *Bosea* sp. PAMC26642, Q8U195.1: *Pyrococcus furiosus* DSM 3638, D7AF64.1: *Geobacter sulfurreducens* KN400, P09832.3: *Escherichia coli* K-12, E1V810.1: *Halomonas elongata* DSM 2581. The blue line indicates NADPH binding motif “GxGxxG” (125-130). G: Glycine residues, X: Any amino acid.

|  |
| --- |


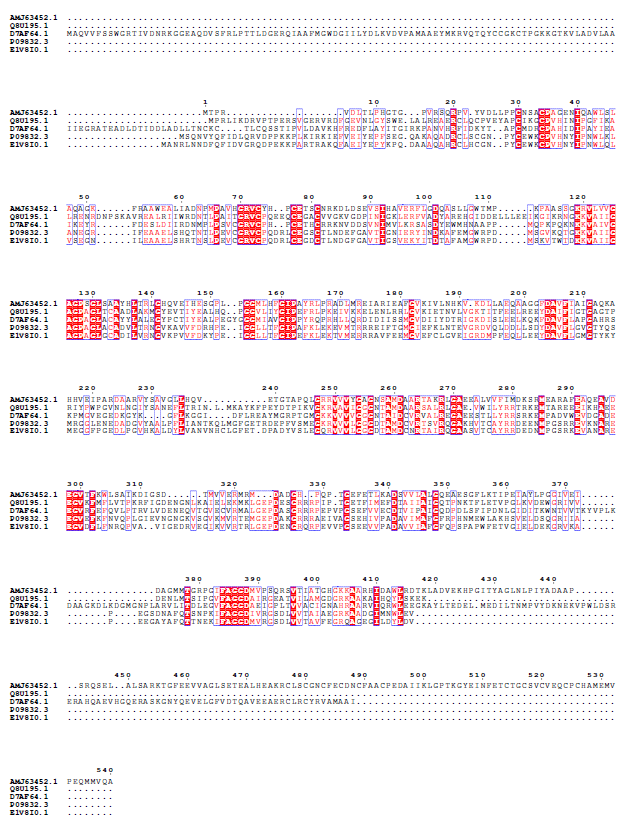


**Supplementary Figure S5.3.** Multiple sequence alignment of glutamine synthetase using Clustal Omega and ESPript 3.0. AMJ60180.1: *Bosea* sp. PAMC26642, P05457.2: *Bradyrhizobium diazoefficiens* USDA 110, P94126.2: *Azorhizobium caulinodans* ORS 571, Q59747.2: *Sinorhizobium meliloti* 1021, P09826.1: *Rhizobium leguminosarum* bv*. Viciae*.


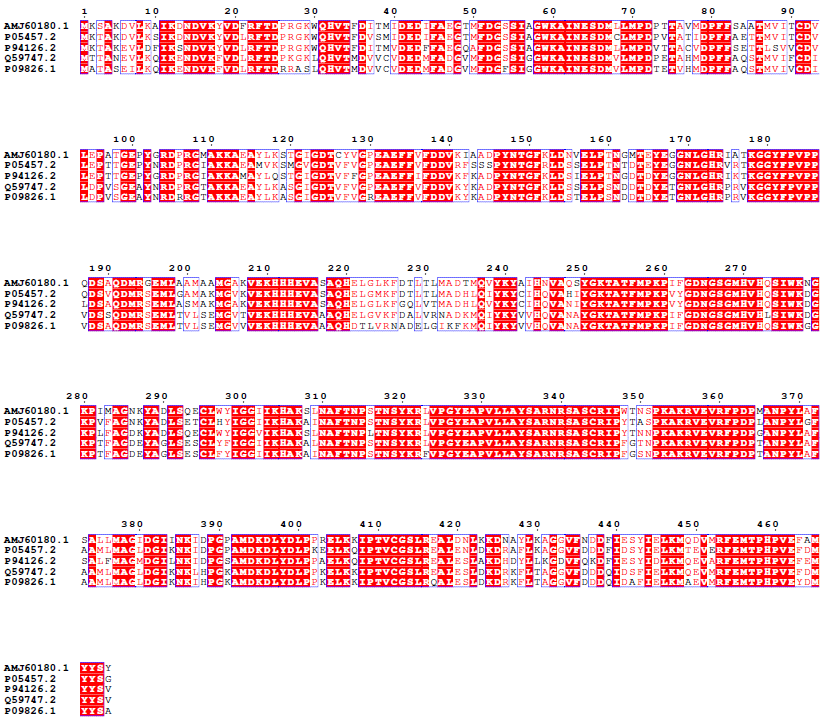


**Supplementary Figure S5.4.** Multiple sequence alignment of nitrate reductase using Clustal Omega and ESPript 3.0. AMJ59270.1: *Bosea* sp. PAMC26642, Q06457.2: *Klebsiella oxytoca,* O33732.2: *Shewanella frigidimarina* NCIMB 400*,* P39458.2: *Synechococcus elongatus PCC 7942 = FACHB-805,* P73448.1: *Synechocystis* sp*.* PCC 6803 *substr. Kazusa*.


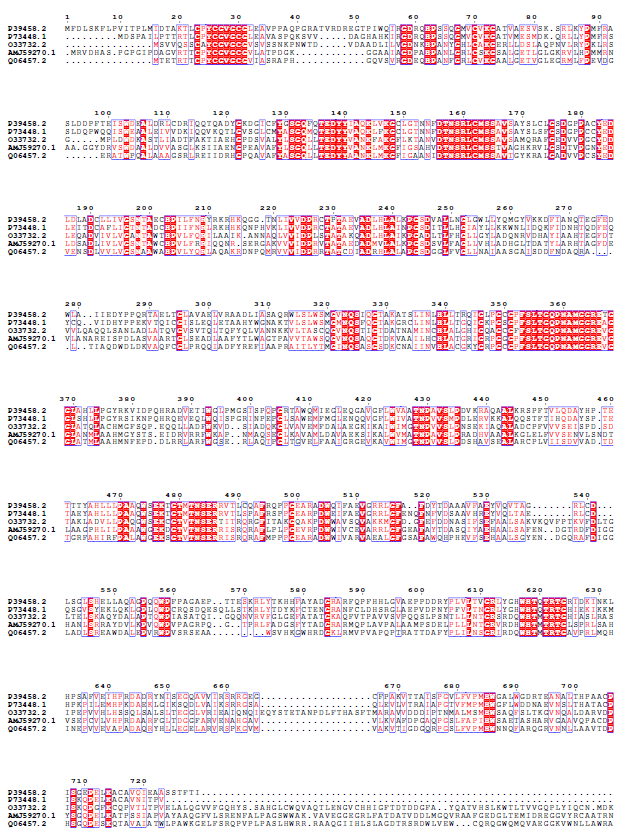

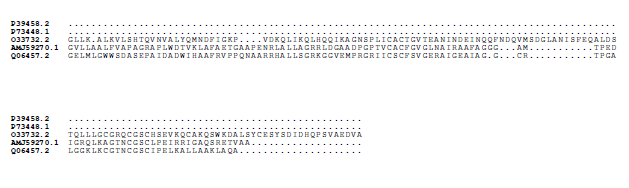


**Supplementary Figure S5.5.** Multiple sequence alignment of nitrite reductase using Clustal Omega and ESPript 3.0. AMJ63395.1: *Bosea* sp. PAMC26642, P39661.1: *Synechococcus elongatus* PCC 7942 *=* FACHB-805, Q51879.1: *Leptolyngbya laminose,* Q73YC1.1: *Mycobacterium avium* subsp. paratuberculosis K-10, P9WJ02.1: *Mycobacterium tuberculosis* CDC1551.


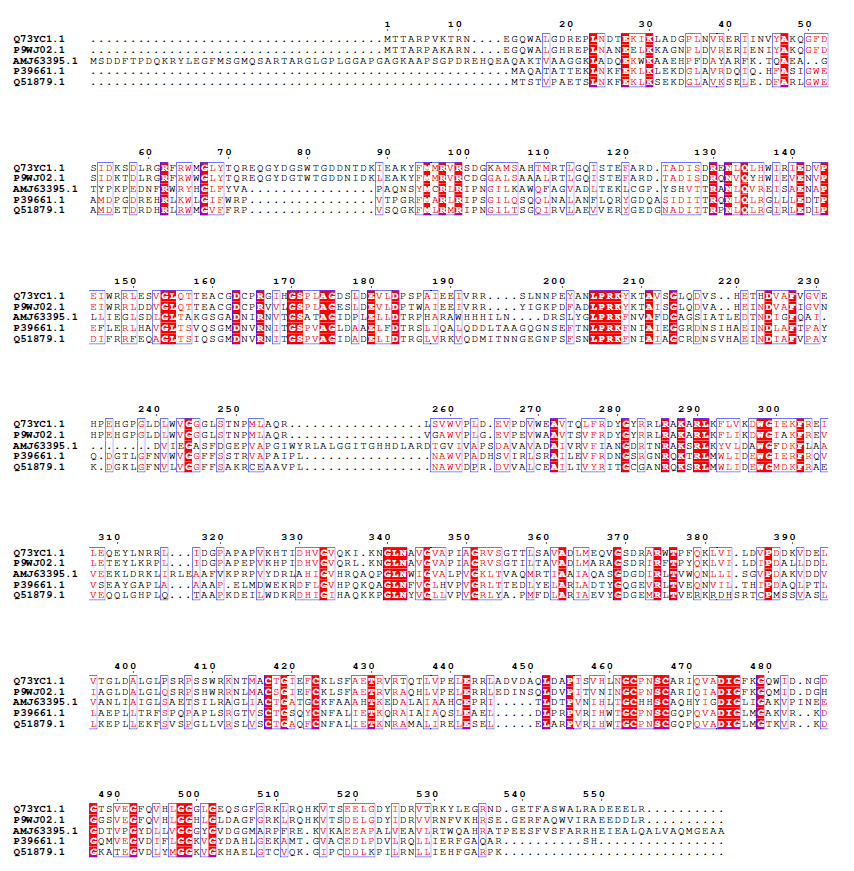


**Supplementary Table S1.** Multiple sequence alignment of nitrate assimilation proteins. The protein sequences were searched against the Swiss-Prot database for the top sequences with the most identities.

| **Proteins** | **Significant alignments** | **Alignment identifies (%)** |
| --- | --- | --- |
|  | **(Accession number)** |  |
| Nitrate reductase | *Klebsiella oxytoca* (Q06457.2) | 421/873(48) |
|  | *Shewanella frigidimarina* NCIMB 400 (O33732.2) | 378/930(41) |
|  | *Synechococcus elongatus* PCC 7942 *= FACHB-805* (P39458.2) | 300/715(42) |
|  | *Synechocystis* sp. PCC 6803 *substr. Kazusa* (P73448.1) | 275/717(38) |
| Nitrite reductase | *Synechococcus elongatus* PCC 7942 *=* FACHB-805 (P39661.1) | 160/475(34) |
|  | [*Leptolyngbya laminosa* (Q51879.1)](https://www.ncbi.nlm.nih.gov/protein/Q51879.1?report=genbank&log$=prottop&blast_rank=2&RID=JSM62RX0013) | 175/481(36) |
|  | [*Mycobacterium avium* subsp. *paratuberculosis* K-10 (Q73YC1.1)](https://www.ncbi.nlm.nih.gov/protein/Q73YC1.1?report=genbank&log$=prottop&blast_rank=9&RID=JSM62RX0013) | 166/553(30) |
|  | [*Mycobacterium tuberculosis* CDC1551 (P9WJ02.1)](https://www.ncbi.nlm.nih.gov/protein/P9WJ02.1?report=genbank&log$=prottop&blast_rank=11&RID=JSM62RX0013) | 165/555(30) |
| Glutamine synthetase | *Bradyrhizobium diazoefficiens* USDA 110 (P05457.2) | 391/468(84) |
|  | *Azorhizobium caulinodans* ORS 571 (P94126.2) | 389/468(83) |
|  | *Sinorhizobium meliloti* 1021 (Q59747.2) | 358/468(76) |
|  | *Rhizobium leguminosarum* bv*. viciae* (P09826.1) | 344/476(72) |
| Glutamate synthase | *Pyrococcus furiosus* DSM 3638 (Q8U195.1) | 143/425(34) |
|  | *Geobacter sulfurreducens* KN400 (D7AF64.1) | 162/506(32) |
|  | *Escherichia coli* K-12 (P09832.3) | 137/429(32) |
|  | *Halomonas elongata* DSM 2581(E1V8I0.1) | 136/418(33) |
| Glutamate dehydrogenase | *Halomonas elongata* DSM 2581 (E1V4J5.1) | 671/1603(42) |
|  | *Pseudomonas aeruginosa* PAO1 (Q9HZE0.1) | 640/1541(42) |
|  | *Mycobacterium tuberculosis* H37Rv (O53203.1) | 617/1577(39) |
|  | *Mycolicibacterium smegmatis* MC2 155 (A0R1C2.1) | 609/1563(39) |

*Nitrate reductase protein of *Bosea* sp. PAMC26642 is 48%, 41%, 42%, and 38% identity with *Klebsiella oxytoca* (Q06457.2), *Shewanella frigidimarina* NCIMB 400 (O33732.2), *Synechococcus elongatus* PCC 7942 *= FACHB-805* (P39458.2), *Synechocystis* sp. PCC 6803 *substr. Kazusa* (P73448.1). Nitrite reductase protein of *Bosea* sp. PAMC26642 is 34%, 36%, 30%, and 30% identity with *Synechococcus elongatus* PCC 7942 *=* FACHB-805 (P39661.1), [*Leptolyngbya laminosa* (Q51879.1)](https://www.ncbi.nlm.nih.gov/protein/Q51879.1?report=genbank&log$=prottop&blast_rank=2&RID=JSM62RX0013), [*Mycobacterium tuberculosis* CDC1551 (P9WJ02.1)](https://www.ncbi.nlm.nih.gov/protein/P9WJ02.1?report=genbank&log$=prottop&blast_rank=11&RID=JSM62RX0013), [*Mycobacterium tuberculosis* CDC1551 (P9WJ02.1)](https://www.ncbi.nlm.nih.gov/protein/P9WJ02.1?report=genbank&log$=prottop&blast_rank=11&RID=JSM62RX0013). glutamine synthetase protein of *Bosea* sp. PAMC26642 is 84%, 83%, 76%, and 72% identity with *Bradyrhizobium diazoefficiens* USDA 110 (P05457.2), *Azorhizobium caulinodans* ORS 571 (P94126.2), *Sinorhizobium meliloti* 1021 (Q59747.2), *Rhizobium leguminosarum* bv*. viciae* (P09826.1). Glutamate synthase protein of *Bosea* sp. PAMC26642 is 34%, 32%, 32%, and 33% identity *Pyrococcus furiosus* DSM 3638 (Q8U195.1), *Geobacter sulfurreducens* KN400 (D7AF64.1), *Escherichia coli* K-12 (P09832.3), *Halomonas elongata* DSM 2581(E1V8I0.1), Glutamate dehydrogenase protein of *Bosea* sp. PAMC26642 is 42%, 42%, 39%, and 39% identity with *Halomonas elongata* DSM 2581 (E1V4J5.1), *Pseudomonas aeruginosa* PAO1 (Q9HZE0.1), *Mycobacterium tuberculosis* H37Rv (O53203.1), *Mycolicibacterium smegmatis* MC2 155 (A0R1C2.1) respectively.

| **Swiss-Prot id** | **NAD-specific glutamate dehydrogenase** | **ACT1 domain** | **ACT2 domain** | **ACT3 domain** | **Bacterial NAD-glutamate dehydrogenase, catalytic domain** | **Glutamate dehydrogenase C terminal** | **Amino acid dehydrogenase-like, N terminal domain** | **NAD(P) binding Rosmann fold** | **N-terminal region of a signal peptide** |
| --- | --- | --- | --- | --- | --- | --- | --- | --- | --- |
| [AMJ62141.1](https://www.ebi.ac.uk/interpro/result/InterProScan/iprscan5-R20241119-110801-0164-69909837-p1m/internal-1732014448055-1491-2/) | 13 - 1614 | 32 - 175 | 407 - 495 | 553 - 638 | 738 - 1232 | 1277 - 1609 | 768 - 953 | - | 1 to 7 |
| E1V4J5.1 | 2 - 1609 | 36 - 179 | 410 - 498 | 555 - 631 | 729 - 1223 | 1268 - 1604 | 758 - 960 | - | - |
| Q9HZE0.1 | 1 - 1617 | 35 - 177 | 407 - 496 | 552 - 629 | 731 - 1225 | 1270 - 1607 | - | 951-1053 | - |
| A0R1C2.1 | 41 - 1587 | 42 - 151 | 370 - 463 | 520 - 582 | 695 - 1197 | 1245 - 1582 | 738 - 934 | - | - |
| O53203.1 | 13 - 1621 | 64 - 176 | 391 - 490 | 550 - 609 | 724 - 1230 | 1278 - 1612 | 768 - 963 | - | - |

**Supplementary Table S2.1** Information on glutamate dehydrogenase protein by InterProScan.

AMJ62141.1: *Bosea* sp. PAMC26642, E1V4J5.1: *Halomonas elongata* DSM 2581, Q9HZE0.1: *Pseudomonas aeruginosa* PAO1,

A0R1C2.1: *Mycolicibacterium smegmatis* MC2 155, O532031: *Mycobacterium tuberculosis* H37Rv.

**Supplementary Table S2.2.** Information on glutamate synthase protein by InterProScan.

| **Swiss-Prot id** | **4Fe-4S ferredoxin -type iron-sulfur binding domain** |
| --- | --- |
| AMJ63452.1 | 474 - 504, 505 - 534 |
| Q8U195.1 | **-** |
| D7AF64.1 | **-** |
| P09832.3 | 38 - 69 |
| E1V8I0.1 | **-** |

AMJ63452.1: *Bosea* sp. PAMC26642, Q8U195.1: *Pyrococcus furiosus* DSM 3638, D7AF64.1:

*Geobacter sulfurreducens* KN400, P09832.3: *Escherichia coli* K-12, E1V810.1: *Halomonas elongata* DSM 2581.

**Supplementary Table S2.3.** Information of glutamine synthetase protein by InterProScan.

| **Swiss-Prot id** | **Glutamine synthetase (GS) beta-grasp domain** | **Glutamine synthetase (GS) catalytic domain** |
| --- | --- | --- |
| AMJ60180.1 | 14 - 99 | 106 - 469 |
| P05457.2 | 14 - 98 | 106 - 469 |
| P94126.2 | 14 - 98 | 106 - 469 |
| Q59747.2 | 14 - 99 | 106 - 469 |
| P09826.1 | 14 - 99 | 106 - 469 |

AMJ60180.1: *Bosea* sp. PAMC26642, P05457.2: *Bradyrhizobium diazoefficiens* USDA 110, P94126.2: *Azorhizobium caulinodans*

ORS 571, Q59747.2: *Sinorhizobium meliloti* 1021, P09826.1: *Rhizobium leguminosarum* bv*. Viciae*.

**Supplementary Table S2.4.** Information on nitrate reductase protein by InterProScan.

| **Swiss-prot id** | **Prokaryotic molybdopterin oxidoreductases 4Fe-4S domain profile** | **MopB_Nitrate-R-NapA-like** | **MopB_CT_Nitrate-R-NapA-like** |
| --- | --- | --- | --- |
| AMJ59270.1 | 14 - 70 | 18 - 587 | 596 - 717 |
| Q06457.2 | 1 - 57 | 5 - 559 | 566 - 687 |
| O33732.2 | 1 - 64 | 5 - 576 | - |
| P39458.2 | 16 - 84 | 20 - 595 | 602 - 723 |
| P73448.1 | 7 - 70 | 11 - 587 | 595 - 714 |

AMJ59270.1: *Bosea* sp. PAMC26642, Q06457.2: *Klebsiella oxytoca,* O33732.2: *Shewanella frigidimarina* NCIMB 400*,*

P39458.2: *Synechococcus elongatus PCC 7942 =FACHB-805,* P73448.1: *Synechocystis* sp*.* PCC 6803 *substr. Kazusa.*

**Supplementary Table S2.5.** Information on nitrite reductase protein by InterProScan.

| **Swiss-Prot id** | **Nitrite/Sulfite reductase ferredoxin-like half-domain** | **Nitrite and sulfite reductase 4Fe-4S domain** |
| --- | --- | --- |
| AMJ63395.1 | 114 - 172, 371 - 436 | 184 - 347, 453 - 568 |
| P39661.1 | 313 - 377, 63 - 125 | 134 - 292, 391-500 |
| Q51879.1 | 61 - 124, 313 - 356 | - |
| Q73YC1.1 | 92 - 152, 338 - 400 | 161 - 313, 413 - 554 |
| P9WJ02.1 | 92 - 152, 338 - 400 | 161 - 314, 412 - 549 |

AMJ63395.1: *Bosea* sp. PAMC26642, P39661.1: *Synechococcus elongatus* PCC 7942 *=* FACHB-805, Q51879.1: *Leptolyngbya laminose,*

Q73YC1.1: *Mycobacterium avium* subsp. paratuberculosis K-10, P9WJ02.1: *Mycobacterium tuberculosis* CDC1551.
